# Racial and Ethnic Discrimination and Hypertension by Educational Attainment Among a Cohort of US Women

**DOI:** 10.1001/jamanetworkopen.2023.44707

**Published:** 2023-11-22

**Authors:** Symielle A. Gaston, Allana T. Forde, Michael Green, Dale P. Sandler, Chandra L. Jackson

**Affiliations:** 1 Epidemiology Branch, National Institute of Environmental Health Sciences, National Institutes of Health, Research Triangle Park, North Carolina; 2 Division of Intramural Research, National Institute on Minority Health and Health Disparities, National Institutes of Health, Bethesda, Maryland; 3 Population Health Sciences Department, Duke University School of Medicine, Durham, North Carolina

## Abstract

**Importance:**

Although understudied, there are likely within-group differences among minoritized racial and ethnic groups in associations between racial and ethnic discrimination (RED) and hypertension risk, as minoritized individuals with higher educational attainment may more frequently encounter stress-inducing environments (eg, professional workplace settings, higher-income stores and neighborhoods) characterized by, for instance, exclusion and antagonism.

**Objectives:**

To investigate educational attainment as a potential effect modifier of associations between RED and hypertension risk among US women; the study hypothesis was that the magnitude of associations would be stronger among participants with higher vs lower educational attainment.

**Design, Setting, and Participants:**

This is a nested case-control study using Sister Study data collected at enrollment (2003-2009) and over follow-up visits until September 2019. Among eligible US Black or African American (hereafter Black), Latina, and non-Hispanic White women without prior hypertension diagnoses, incidence density sampling was performed to select self-reported hypertension cases that developed over a mean (SD) follow-up 11 (3) years. Data were analyzed August 2022 to February 2023.

**Exposures:**

Participants reported lifetime everyday (eg, unfair treatment at a business) and major (eg, mistreatment by police) RED via a self-administered questionnaire.

**Main Outcome and Measures:**

Adjusting for sociodemographic characteristics, conditional logistic regression was used to estimate odds ratios (ORs) and 95% CIs for associations between RED and hypertension by educational attainment category at baseline (college or higher, some college, and high school or less) within racial and ethnic groups.

**Results:**

Among 5179 cases (338 [6.5%] Black; 200 [3.9%] Latina; and 4641 [89.6%] non-Hispanic White) and 10:1 race and ethnicity– and age-matched control participants with a mean (SD) age of 55 (9) years at enrollment, half (49.9%) of women reported attaining college or higher education, and Black women with college or greater education had the highest burden of RED (eg, 83% of case participants with college or higher education reported everyday RED compared with 64% of case participants with high school or less education). Everyday RED was associated with higher hypertension risk among Black women with college or higher education (OR, 1.56 [95% CI, 1.06-2.29]) but not among Black women with some college (OR, 0.72 [95% CI, 0.47-1.11]), with evidence of both multiplicative and additive interaction. Results for Black women with high school or less education suggested increased risk, but confidence intervals were wide, and the result was not statistically significant but may be clinically significant (OR, 1.89 [95% CI, 0.83-4.31]). Educational attainment was not a modifier among other racial and ethnic groups or for associations with major RED.

**Conclusions and Relevance:**

In this nested case-control study of RED and hypertension risk, chronic or everyday RED-associated hypertension disproportionately affected Black women with the highest levels of educational attainment.

## Key Points

**Question:**

Does educational attainment modify associations between racial and ethnic discrimination and hypertension risk within groups of Black or African American, Latina, and non-Hispanic White US women?

**Findings:**

In this nested case-control analysis including 5179 women with hypertension and 10:1 race and ethnicity– and age-matched control participants, educational attainment modified associations between perceived everyday racial and ethnic discrimination and higher hypertension risk only among Black or African American women. Black or African American women with a Bachelor’s degree or higher most frequently reported everyday racial and ethnic discrimination and had higher associated risk than counterparts with some college.

**Meaning:**

In this study, racial and ethnic discrimination–related hypertension risk appeared to disproportionately affect Black or African American women with the highest levels of education.

## Teaser

This case-control study investigates educational attainment as a potential effect modifier of associations between racial and ethnic discrimination and hypertension risk among US women.

## Introduction

Hypertension is a highly prevalent risk factor for cardiovascular disease, the leading cause of death among women in the United States.^1,2^ Among US women, hypertension prevalence is highest for non-Hispanic Black or African American (hereafter Black) women. Beyond other identified risk factors, likely contributors to hypertension among Black women include stressors related to intersectionality or membership in multiple marginalized groups, namely marginalization based on gender coupled with racial and ethnic discrimination.^3,4,5,6,7,8,9^ Psychosocial stress related to experiencing discrimination can activate biologic stress response pathways as well as contribute to maladaptive health behaviors (eg, suboptimal diet) that are risk factors for hypertension.^10,11,12^ Chronic discrimination may also contribute to long-term physiological wear and tear or increased allostatic load and, for instance, arousal during sleep periods—another risk factor for hypertension.^11,13,14,15^

Associations between experiencing racial and ethnic discrimination (RED) and hypertension likely vary by educational attainment within racial and ethnic groups.^6,8,9,16^ Reports of experiencing racial and ethnic discrimination are more common among minoritized racial and ethnic groups, particularly Black persons.^9,10,17,18^ There is also within-group variation in discrimination experiences.^9^ For instance, Black adults with higher educational attainment often work in environments where Black people were historically excluded and/or are currently underrepresented due to inequitable hiring practices^19^ and therefore may be more likely to experience subtle forms of discrimination.^9,20^ Furthermore, higher education often yields higher incomes, which increases opportunity to live in salubrious environments. However, prior studies indicate—based on the diminishing returns hypothesis—that racially or ethnically minoritized neighbors may not experience the same benefits and access to resources as their non-Hispanic White (hereafter White) counterparts due to factors such as structural and interpersonal racism.^9,19,21,22^ Hence, racially or ethnically minoritized adults attaining higher vs lower education may be more frequently exposed to multiple forms of discrimination, potentially resulting in an exacerbated risk of discrimination-related hypertension.

Although relationships between socioeconomic status discrimination and a risk factor for hypertension, C-reactive protein, have been shown only among Black persons in a multiracial sample,^23^ to our knowledge, empirical studies in the United States have not yet investigated educational attainment as a modifier of associations between RED specifically and hypertension risk while also considering potential differences within racial and ethnic groups.^6,24,25,26,27,28,29,30,31^ To overcome these literature gaps, we aimed to investigate race and ethnicity as well as educational attainment as modifiers of associations between RED and hypertension risk among a cohort of Black, Latina, and White US women. We hypothesized that (1) the highest prevalence of RED would be among Black women who attained a Bachelor’s degree or higher; (2) the highest incidence of hypertension would be among Black women irrespective of educational attainment; (3) RED would be associated with a higher risk of hypertension; and (4) while there would be no difference in associations by educational attainment among White women, there would be stronger associations among Black and Latina women with higher vs lower educational attainment.

## Methods

### Data Source: The Sister Study

We used data (release 9.1 with follow-up through September 30, 2019) from the Sister Study, which is described in detail elsewhere.^32^ Briefly, the Sister Study is an ongoing cohort study of 50 884 US (including Puerto Rico) women aged 35 to 74 years at enrollment (2003-2009) who had a sister diagnosed with breast cancer but had no prior breast cancer diagnosis themselves. Women provided written informed consent prior to participation. The Sister Study protocol was approved and is overseen by the National Institutes of Health institutional review board, which waived approval for this secondary data analysis. This study followed the Strengthening the Reporting of Observational Studies in Epidemiology (STROBE) reporting guideline.

### Analytic Sample

We excluded Sister Study participants who did not meet eligibility criteria (eg, no prior hypertension diagnosis) in a stepwise manner (eFigure 1 in Supplement 1), which resulted in 26 846 eligible participants without prior hypertension at the time of the RED assessment. Compared with ineligible Black, Latina, and White participants without prior hypertension diagnosis, eligible participants were younger, more likely to identify as White, and more likely to report higher socioeconomic status as well as healthier behaviors and recommended weight (eTable 1 in Supplement 1).

### Exposure Assessment: RED

We assessed lifetime RED using data collected from a questionnaire self-administered during 2008 to 2012 that was adapted from The Everyday Discrimination Scale and prior literature.^17,33^ Women reported whether they ever experienced any of 3 forms of everyday RED (ie, unfair treatment at a store, restaurant, or other place of business; treated as less intelligent, worthy, or honest; or experienced people acting as if they are afraid of you due to your race or ethnicity) and any of 3 forms of major RED (ie, treated unfairly in home renting, buying, or mortgaging; treated unfairly in being stopped, searched, or threatened by the police; or treated unfairly in job hiring, promotion, or firing due to your race or ethnicity) during their lifetime. Consistent with prior studies,^10,23,34^ we dichotomized everyday and major RED (ever or yes to any vs never or no to all). We assessed everyday RED and major RED separately as well as either everyday or major RED vs neither.

### Case Definition: Incident Hypertension

Over follow-up data collection until September 30, 2019, participants provided a yes or no response to, “Has a doctor or other health professional told you that you had hypertension or high blood pressure?” If participants responded yes, they were considered a case; they also provided the month and year of diagnosis, which allowed for calculation of age at diagnosis.

### Potential Confounders and Effect Modifiers

A priori potential confounders were self-reported at baseline and are detailed in Table 1. Sociodemographic characteristics included age, self-identified race and ethnicity defined using Office of Management and Budget categories (Black, Latina [any race and Hispanic/Latina ethnicity], or White),^38^ longest lived region of residence as an adult, marital status, educational attainment, current employment, and annual household income. Health behaviors included smoking status, alcohol consumption in the past 12 months, physical activity,^35^ diet,^36^ and sleep.^10,37^ Clinical characteristics included body mass index category and menopausal status. We identified confounders to retain and potential mediators through construction of directed acyclic graphs (eFigure 2 in Supplement 1). Educational attainment (high school or less, some college, and college or higher) was also considered a potential effect modifier.

**Table 1.** Case and Control Participant Characteristics, Overall and by Educational Attainment, With Data From the Sister Study

Characteristic^a^	Participants, %							
	Total		≥College		Some college		≤High school	
	Hypertension cases (n = 5179)	Controls (n = 51 783)	Hypertension cases (n = 2586)	Controls (n = 29 733)	Hypertension cases (n = 1763)	Controls (n = 15 516)	Hypertension cases (n = 830)	Controls (n = 6534)
**Sociodemographic characteristics**								
Age, mean (SD), y	55.2 (8.8)	56.1 (8.6)	55.1 (8.8)	55.6 (8.5)	54.6 (8.7)	56.5 (8.5)	56.4 (8.7)	57.8 (8.8)
Age category								
≤55 y	51.6	47.3	50.9	49.2	55.2	46.8	46.3	40.1
>55 y	48.4	52.7	49.1	50.8	44.8	53.2	53.7	59.9
Race and ethnicity								
Black or African American (non-Hispanic)	6.5	6.5	7.9	7.1	6.0	6.6	3.4	3.8
Hispanic or Latina	3.9	3.8	2.9	3.3	4.9	4.3	4.5	5.5
White (non-Hipanic)	89.6	89.6	89.2	89.7	89.1	89.1	92.2	90.7
Region of residence lived longest as an adult								
Northeast	18.1	19.8	19.6	20.7	15.3	17.4	19.4	20.9
Midwest	30.5	28.2	28.2	25.7	32.2	30.8	34.5	33.2
South	30.5	27.9	31.2	28.4	30.9	27.3	27.7	27.0
West	19.1	22.5	19.5	23.6	19.8	22.9	16.4	16.9
Puerto Rico or outside US and Puerto Rico	1.8	1.6	1.6	1.5	1.9	1.6	2.0	2.0
Marital status								
Married or living as married	76.0	77.5	73.9	76.3	76.1	78.1	82.3	81.2
Single or never married	5.3	4.6	7.2	5.9	4.2	3.0	2.2	2.0
Divorced, separated, or widowed	18.7	18.0	19.0	17.7	19.7	18.9	15.5	16.8
Educational attainment								
≥College	49.9	57.4	100.0	100.0	0	0	0	0
Some college or technical degree	34.0	30.0	0	0	100.0	100.0	0	0
≤High school	16.0	12.6	0	0	0	0	100.0	100.0
Currently employed^b^	68.1	65.5	71.4	68.7	67.4	63.5	59.2	55.4
Annual household income, $								
<20 000	3.6	3.0	1.5	1.6	5.0	4.2	7.5	6.9
20 000-49 999	21.6	18.5	14.1	12.2	26.0	24	35.5	33.8
50 000-99 999	43.1	41.2	41.8	38.8	45.2	44.8	42.8	43.1
≥100 000	31.6	37.4	42.6	47.4	23.8	27.1	14.2	16.2
**Health behaviors**								
Smoking status								
Current	8.4	6.1	5.3	4.1	10.6	8.2	13.5	10.7
Former	37.5	36.5	34.4	34.1	39.8	39.4	42.2	40.2
Never	54.1	57.4	60.3	61.8	49.6	52.5	44.3	49.2
Alcohol consumption (past 12 mos)								
Current, ≥2 drinks/d	5.9	4.8	6.4	5.0	5.0	4.7	6.1	4.5
Current, <1 to <2 drinks/d	77.1	79.1	79.5	81.4	77.4	77.5	68.7	72.2
Never/former	17.0	16.1	14.1	13.6	17.8	17.8	25.2	23.3
Physical activity, log-METs-h/wk mean (SD)^c^	3.7 (0.7)	3.8 (0.6)	3.7 (0.6)	3.8 (0.6)	3.7 (0.7)	3.8 (0.6)	3.8 (0.7)	3.8 (0.7)
Healthy Eating Index score, mean (SD)^d^	71.3 (9.7)	73.2 (9.4)	73.0 (9.1)	74.4 (8.8)	70.0 (9.9)	72.0 (9.7)	69.0 (10.3)	70.8 (10.1)
Sleep score, mean (SD)^e^	1.1 (1.2)	1.0 (1.2)	1.0 (1.2)	0.9 (1.1)	1.2 (1.3)	1.1 (1.2)	1.3 (1.3)	1.2 (1.3)
**Clinical characteristics**								
BMI category								
Underweight (<18.5)	0.9	1.4	1.0	1.6	0.8	1.3	0.7	1.2
Recommended weight (18.5-24.9)	34.7	49.1	39.4	54.2	30.6	43.3	28.7	39.7
Overweight (25.0-29.9)	35.7	31.4	32.9	29.1	37.7	33.4	40.1	37.4
Obesity (≥30.0)	28.7	18.0	26.6	15.1	30.9	22.0	30.5	21.7
Postmenopausal	69.6	69.8	66.1	67.6	65.1	71.3	71.6	76.6
**Racial and ethnic discrimination**								
Everyday								
Yes	9.7	9.3	11.7	10.4	9.0	8.9	4.7	4.9
No	90.3	90.7	88.3	89.6	91.0	91.1	95.3	95.1
Major								
Yes	6.5	5.8	7.8	5.7	5.7	94.6	4.2	2.9
No	93.5	94.2	92.2	93.3	94.3	5.4	95.8	97.1
Everyday or major								
Yes	12.1	11.1	14.2	12.4	11.2	10.7	7.2	6.2
No	87.9	88.9	85.8	87.6	88.8	89.3	92.8	93.8

Abbreviations: BMI, body mass index (calculated as weight in kilograms divided by height in meters squared); METs, metabolic equivalent of tasks.

^a^ Missingness: less than 0.01% for marital status and BMI category.

^b^ Proportion employed is calculated as: No. employed / (No. employed + unemployed + homemaker + student + retired).

^c^ Physical activity is measured continuously as the log-transformed total MET hours per week engaged in sports or exercise and daily activities.^35^

^d^ Diet is assessed using Healthy Eating Index scores that range from 0 to 100, with higher scores indicating a healthier diet.^36^

^e^ Sleep score is a summary score for 6 poor sleep dimensions.^10,37^ Sleep score ranges from 0 to 6. Participants were assigned a value of 1 for each if they reported experiencing the following: (1) habitual short [<7-hour] or long [>9-hour] sleep duration (vs recommended [7- to 9-hour]); (2) inconsistent weekly sleep patterns, defined as consistent [could vary day-by-day but were stable from week-to-week] or inconsistent wake-up times and bedtimes during the prior 6 weeks (yes vs no); (3) sleep debt, defined as 2-hour or greater difference between average longest and shortest sleep duration; (4) frequent napping (≥3 d/wk vs <3 d/wk); (5) difficulty falling asleep, defined as taking more than 30 minutes vs 30 or fewer minutes to fall asleep on average; and (6) difficulty staying asleep, defined as waking up 3 or more times per night 3 or more nights/week vs less than 3 times per night less than 3 nights/week.

### Statistical Analysis

Within the pool of 26 846 participants, we selected all cases of hypertension (n = 5179) and performed incidence density sampling, selecting 10 race and ethnicity– and age-matched control participants per case given the unbiased risk estimates identified using 10:1 matching in prior literature.^39^ Black women are more likely to develop hypertension at earlier ages; therefore, this approach addresses the potential for immortal time bias related to racial and ethnic differences in age of hypertension onset.^40,41^ Matching was performed by randomly selecting control participants from the pool of eligible race and ethnicity–matched individuals (except the index case participant) who attained at least the age at hypertension diagnosis of the case participant. Therefore, it was possible for a case participant who developed hypertension at an older age to serve as a control participant for a case participant who developed hypertension at a younger age and for a control participant to match to multiple case participants.

We then described characteristics of case and control participants overall, by educational attainment, and by educational attainment within each racial and ethnic group using frequencies and percentages for categorical variables and means and SDs for continuous variables. Using conditional logistic regression that approximates risk given incidence density sampling in nested case-control analyses,^39,42^ we estimated odds ratios (ORs) and 95% CIs for associations between discrimination (everyday, major, and either form of RED) and hypertension risk overall and within each racial and ethnic group. Following suggested guidelines for assessing effect modification (EM),^42^ we estimated RED × educational attainment categories associated with hypertension case status. Models were also stratified by educational attainment. Additive EM was assessed by estimating the relative excess risk due to interaction (RERI), and multiplicative EM was assessed with cross-product terms.^42^ If data suggested RERI, we also estimated the proportion attributable to interaction (AP), which represents the proportion of hypertension risk in the doubly exposed group that is attributable to interaction. Models were adjusted for race and ethnicity (in models among the overall population), age in 5-year increments, longest lived region of residence, and current employment. All analyses were performed using SAS version 9.4 for Windows (SAS Institute), and a 2-sided *P* < .05 determined statistical significance.

In sensitivity analyses, we additionally adjusted all models for potential mediators, which allowed for estimation of the direct effect (rather than the total effect estimated in the main analysis)^43^: annual household income and health behaviors (smoking status, alcohol consumption, diet, physical activity, and sleep). Lastly, we further adjusted the original models for birthplace among Latina participants, the only group with significant variation in birthplace, which could impact discrimination experiences.^10^

## Results

### Study Population Characteristics

The mean (SD) age at baseline was 55 (9) years. Over a mean (SD) follow-up of 11 (3) years, 5179 hypertension cases developed (338 [6.5%] among Black women; 200 [3.9%], Latina women; 4641 [89.6%], White women) (Table 1; eTables 2-4 in Supplement 1). Incidence was highest among Black women (265 cases per 10 000 person-years) followed by Latina (178 per 10 000 person-years) and White (174 per 10 000 person-years) women. Due to incidence density sampling, which allows (1) case participants who develop hypertension at older ages to serve as control participants to those who develop hypertension at earlier ages and (2) a control participant to serve as a control participant for multiple case participants, cases were successfully matched to 51 783 controls (6.5% Black; 3.8% Latina; 89.6% White). Black women had the highest educational attainment irrespective of case-control status (60% of case participants and 62% of control participants reporting college or higher) compared with White (50% of case participants and 57% of control participants reporting college or higher) and Latina women (38% of case participants and 49% of control participants reporting college or higher). Black women were the most likely to report RED (Figure), and Black women with college or higher education more frequently reported all forms of RED than within–race and ethnicity counterparts with lower educational attainment (eg, everyday RED prevalence for college or higher, some college, and high school or less among case participants was 83%, 66%, and 64%, respectively, and 77%, 74%, and 51%, respectively, among control participants).

**Figure.**
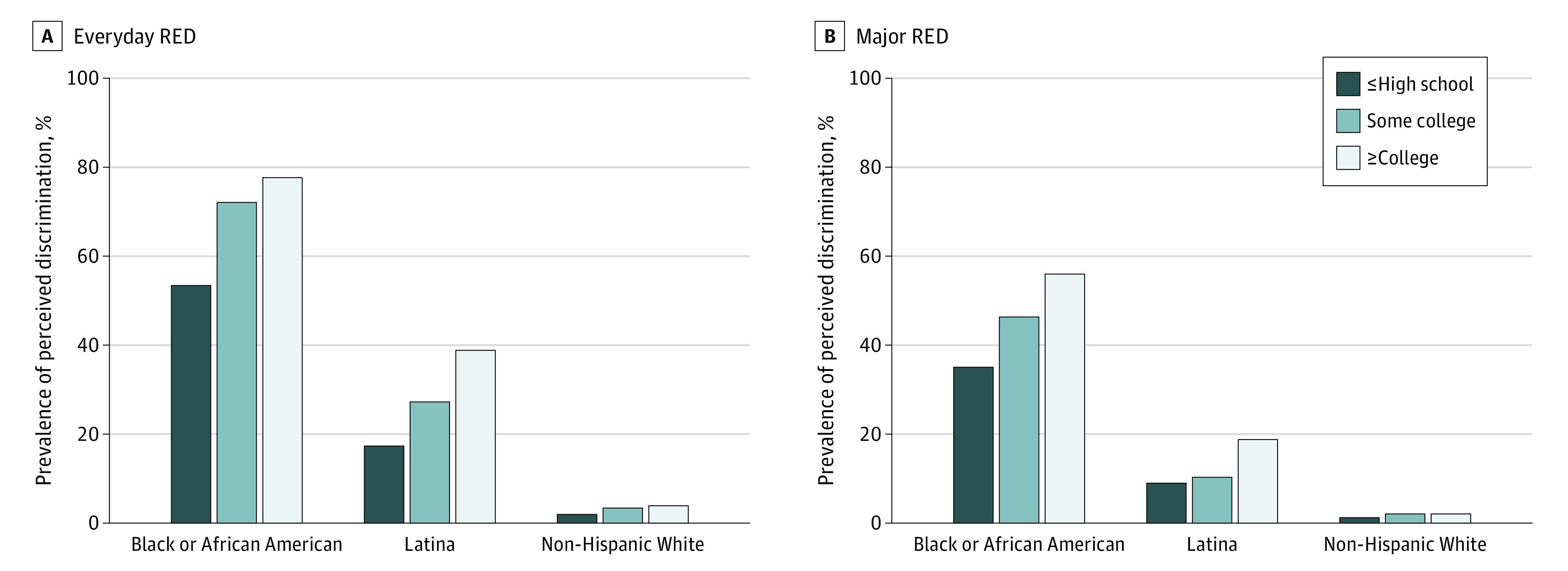
Prevalence of Perceived Everyday and Major Racial and Ethnic Discrimination (RED) by Educational Attainment Within Racial and Ethnic Groups

### RED and Hypertension, Overall and by Race and Ethnicity

Each form of RED was associated with a higher risk of hypertension among the overall population. There was little suggestion of EM by race and ethnicity (eTable 5 in Supplement 1).

### Everyday RED and Hypertension, by Educational Attainment Within Racial and Ethnic Groups

There was additive and multiplicative EM by educational attainment only among Black women (RERI for some college vs college or higher, −1.08 [95% CI, −2.16 to −0.01]; ratio of ORs for some college vs college or higher, 0.46 [95% CI, 0.26 to 0.83]) (Table 2). Compared with Black women with college or higher education and without perceived everyday RED, Black women with college or higher education and perceived everyday RED had higher risk of hypertension (OR, 1.56 [95% CI, 1.06 to 2.29]). There was no association between perceived everyday RED and hypertension risk among Black women who completed some college (OR, 0.72 [95% CI, 0.47-1.11]). The AP for some college vs college or higher education was −0.79 (95% CI, −1.52 to −0.06); 79% of the lower risk of hypertension among Black women with some college who reported everyday RED was due to EM by educational attainment. Among Black women with high school or less education, the OR was greater than 1, but the result was not statistically significant because of a wide 95% CI (OR, 1.89 [95% CI, 0.83 to 4.31]) and, based on tests for multiplicative and additive EM, did not differ from associations among Black women with college or higher education.

**Table 2.** Odds Ratios for Associations Between Perceived Everyday Racial and Ethnic Discrimination and Hypertension Incidence Overall and Within Racial and Ethnic Groups, Stratified by Educational Attainment^a^

Educational attainment	No everyday racial and ethnic discrimination				Everyday racial and ethnic discrimination				Everyday racial and ethnic discrimination within strata of educational attainment	
	Cases, No.	Controls, No.	OR (95% CI)	*P* value	Cases, No.	Controls, No.	OR (95% CI)	*P* value	OR (95% CI)	*P* value
**Overall** ^b^										
≥College	2283	26 628	1 [Reference]	NA	303	3105	1.20 (1.04-1.39)	.01	1.20 (1.04-1.39)	.01
Some college or technical degree	1605	14 136	1.35 (1.27-1.45)	<.001	158	1380	1.44 (1.19-1.74)	<.001	1.06 (0.88-1.29)	.52
≤High school	791	6216	1.55 (1.42-1.69)	<.001	39	318	1.55 (1.10-2.18)	.01	1.00 (0.70-1.41)	.98
**Black or African American** ^c^										
≥College	34	485	1 [Reference]	NA	170	1621	1.56 (1.06-2.29)	.02	1.56 (1.06-2.29)	.02
Some college or technical degree	36	272	1.89 (1.15-3.11)	.01	70	753	1.37 (0.89-2.10)	.15	0.72 (0.47-1.11)	.14
≤High school	10	121	1.21 (0.58-2.54)	.61	18	128	2.29 (1.24-4.23)	.01	1.89 (0.83-4.31)	.13
**Latina** ^d^										
≥College	48	588	1 [Reference]	NA	28	379	0.94 (0.57-1.55)	.81	0.94 (0.57-1.55)	.81
Some college or technical degree	64	488	1.67 (1.12-2.49)	.01	23	178	1.78 (1.05-3.03)	.03	1.07 (0.64-1.79)	.80
≤High school	34	296	1.51 (0.94-2.43)	.09	3	64	0.65 (0.20-2.18)	.49	0.43 (0.13-1.46)	.18
**Non-Hispanic White** ^e^										
≥College	2201	27 756	1 [Reference]	NA	105	1105	1.12 (0.91-1.38)	.28	1.12 (0.91-1.38)	.28
Some college or technical degree	1505	13 376	1.34 (1.25-1.44)	<.001	65	449	1.75 (1.34-2.28)	<.001	1.31 (1.00-1.71)	.05
≤High school	747	5799	1.57 (1.44-1.72)	<.001	18	126	1.65 (1.00-2.71)	.05	1.05 (0.63-1.73)	.85

Abbreviations: NA, not applicable; OR, odds ratio.

^a^ Conditional logistic regression models are adjusted for age in 5-year increments, race and ethnicity (in the overall population; Black or African American [non-Hispanic], Latina, or non-Hispanic White), longest lived region of residence (Northeast, Midwest, South, West, Puerto Rico, or outside the US and Puerto Rico), and current employment (yes or no). Case and control participants were race and ethnicity– and age- matched.

^b^ Measures of effect modification on the additive scale in the overall population were as follows: relative excess risk due to interaction (RERI) for some college vs college or higher, −0.11 (95% CI, −0.41 to 0.18); RERI for high school or less vs college or higher, −0.21 (95% CI, −0.76 to 0.34). Measures of effect modification on the multiplicative scale in the overall population were as follows: ratio of ORs for some college vs college or higher, 0.89 (95% CI, 0.57 to 1.19); ratio of ORs for high school or less vs college or higher, 0.83 (95% CI, 0.72 to 1.10).

^c^ Measures of effect modification on the additive scale among Black or African American women: RERI for some college vs college or higher, −1.08 (95% CI, −2.16 to −0.01); RERI for high school or less vs college or higher, 0.51 (95% CI, −0.92 to 1.95). Measures of effect modification on the multiplicative scale among Black or African American women: ratio of ORs for some college vs college or higher, 0.46 (95% CI, 0.26 to 0.83); ratio of ORs for high school or less vs college or higher, 1.21 (95% CI, 0.49 to 3.00).

^d^ Measures of effect modification on the additive scale among Latina women: RERI for some college vs college or higher_, _0.17 (95% CI, −0.83 to 1.18); RERI for high school or less vs college or higher, −0.80 (95% CI, −1.95 to 0.34). Measures of effect modification on the multiplicative scale among Latina women: ratio of ORs for some college vs college or higher, 1.13 (95% CI, 0.56 to 2.32); ratio of ORs for high school or less vs college or higher, 0.46 (95% CI, 0.12 to 1.71).

^e^ Measures of effect modification on the additive scale among non-Hispanic White women: RERI for some college vs college or higher, 0.29 (95% CI, −0.23 to 0.81); RERI for high school or less vs college or higher, −0.05 (95% CI, −0.90 to 0.81). Measures of effect modification on the multiplicative scale among non-Hispanic White women: ratio of ORs for some college vs college or higher, 1.17 (95% CI, 0.83 to 1.63); ratio of ORs for high school or less vs college or higher, 0.94 (95% CI, 0.54 to 1.61).

### Major RED and Hypertension, by Educational Attainment Within Racial and Ethnic Groups

Data generally suggested perceived major RED was associated with higher risk of hypertension. However, there was no evidence of additive or multiplicative EM by educational attainment within racial and ethnic groups (Table 3).

**Table 3.** Odds Ratios for Associations Between Perceived Major Racial and Ethnic Discrimination and Hypertension Incidence Overall and Within Racial and Ethnic Groups, Stratified by Educational Attainment^a^

Educational attainment	No major racial and ethnic discrimination				Major racial and ethnic discrimination				Major racial and ethnic discrimination within strata of educational attainment	
	Cases, No.	Controls, No.	OR (95% CI)	*P* value	Cases, No.	Controls, No.	OR (95% CI)	*P* value	OR (95% CI)	*P* value
**Overall** ^b^										
≥College	2383	27 752	1 [Reference]	NA	203	1981	1.32 (1.11-1.56)	<.001	1.32 (1.11-1.56)	<.001
Some college or technical degree	1662	14 683	1.35 (1.26-1.44)	<.001	101	833	1.57 (1.26-1.97)	<.001	1.17 (0.93-1.46)	.18
≤High school	795	6346	1.53 (1.40-1.66)	<.001	35	188	2.42 (1.67-3.50)	<.001	1.58 (1.09-2.31)	.02
**Black/African American** ^c^										
≥College	83	935	1 [Reference]	NA	121	1171	1.28 (0.95-1.73)	.10	1.28 (0.95-1.73)	.10
Some college or technical degree	59	554	1.24 (0.87-1.76)	.24	47	471	1.19 (0.82-1.74)	.37	0.96 (0.64-1.45)	.86
≤High school	13	167	0.94 (0.51-1.75)	.85	15	82	2.48 (1.35-4.56)	<.001	2.64 (1.18-5.89)	.02
**Latina** ^d^										
≥College	63	785	1 [Reference]	NA	13	182	0.97 (0.52-1.83)	.94	0.97 (0.52-1.83)	.94
Some college or technical degree	73	604	1.58 (1.10-2.25)	.01	14	62	3.54 (1.83-6.83)	<.001	2.24 (1.17-4.31)	.02
≤High school	35	326	1.46 (0.93-2.29)	.10	2	34	0.77 (0.18-3.31)	.72	0.53 (0.12-2.32)	.40
**Non-Hispanic White** ^e^										
≥College	2201	25 555	1 [Reference]	NA	105	1105	1.30 (1.00-1.67)	.05	1.30 (1.00-1.67)	.05
Some college or technical degree	1505	13 376	1.35 (1.26-1.45)	<.001	65	449	1.64 (1.17-2.29)	<.001	1.22 (0.87-1.70)	.25
≤High school	747	5799	1.56 (1.43-1.70)	<.001	18	126	3.09 (1.83-5.21)	<.001	1.98 (1.17-3.35)	.01

Abbreviations: NA, not applicable; OR, odds ratio.

^a^ Conditional logistic regression models are adjusted for age in 5-year increments, race and ethnicity (in the overall population; Black or African American [non-Hispanic], Latina, or non-Hispanic White), longest lived region of residence (Northeast, Midwest, South, West, Puerto Rico or outside the US and Puerto Rico), and current employment (yes or no). Cases and controls were race and ethnicity– and age-matched.

^b^ Measures of effect modification on the additive scale in the overall population: relative excess risk due to interaction (RERI) for some college vs college or higher, −0.09 (95% CI, −0.48 to 0.29); RERI for high school or less vs college or higher, −0.57 (95% CI, −0.34 to 1.48). Measures of effect modification on the multiplicative scale in the overall population: ratio of ORs for some college vs college or higher, 0.88 (95% CI, 0.68 to 1.15); ratio of ORs for high school or less vs college or higher, 1.20 (95% CI, 0.80 to 1.80).

^c^ Measures of effect modification on the additive scale among Black or African American women: RERI for some college vs college or higher, −0.33 (95% CI, −0.96 to 0.30); RERI for high school or less vs college or higher, 1.26 (95% CI, −0.27 to 2.78). Measures of effect modification on the multiplicative scale among Black or African American women: ratio of ORs for some college vs college or higher, 0.75 (95% CI, 0.45 to 1.25); ratio of ORs for high school or less vs college or higher, 2.05 (95% CI, 0.87 to 4.86).

^d^ Measures of effect modification on the additive scale among Latina women: RERI for some college vs college or higher, 1.99 (95% CI, −0.27 to 4.24); RERI for high school or less vs college or higher, −0.67 (95% CI, −2.09 to 0.76). Measures of effect modification on the multiplicative scale among Latina women: ratio of ORs for some college vs college or higher, 2.30 (95% CI, 0.93 to 5.71); ratio of ORs for high school or less vs college or higher, 0.54 (95% CI, 0.11 to 2.74).

^e^ Measures of effect modification on the additive scale among non-Hispanic White women: RERI for some college vs college or higher, 0.00 (95% CI, −0.64 to 0.63); RERI for high school or less vs college or higher, 1.23 (95% CI, −0.41 to 2.88). Measures of effect modification on the multiplicative scale among non-Hispanic White women: ratio of ORs for some college vs college or higher, 0.94 (95% CI, 0.62 to 1.43); ratio of ORs for high school or less vs college or higher, 1.53 (95% CI, 0.85 to 2.75).

### Everyday or Major RED and Hypertension by Educational Attainment Within Racial and Ethnic Groups

There was additive and multiplicative EM by educational attainment only among Black women (RERI for some college vs college or higher, −1.60 [95% CI, −3.15 to −0.04]; ratio of ORs for some college vs college or higher, 0.37 [95% CI, 0.19 to 0.72) (Table 4). Among Black women with some college, perceived everyday or major RED was not associated with hypertension risk (OR, 0.72 [95% CI, 0.46 to 1.14]), but among Black women with college or higher education, women who reported vs did not report either everyday or major RED had approximately 2-fold higher odds of hypertension (OR, 1.94 [95% CI, 1.21 to 3.10]). The AP for some college vs college or higher education was −0.93 (95% CI, −1.70 to −0.15); 93% of the lower hypertension risk among Black women with some college who reported either everyday or major RED was due to EM by educational attainment.

**Table 4.** Odds Ratios for Associations Between Either Perceived Everyday or Major Racial and Ethnic Discrimination and Hypertension Incidence Overall and Within Racial and Ethnic Groups, Stratified by Educational Attainment

Educational attainment^a^	No everyday or major racial and ethnic discrimination				Either everyday or major racial and ethnic discrimination				Everyday or major racial and ethnic discrimination within strata of educational attainment	
	No. of cases	No. of controls	OR (95% CI)	*P* value	No. of cases	No. of controls	OR (95% CI)	*P* value	OR (95% CI)	*P* value
**Overall** ^b^										
≥College	2218	26 045	1 [Reference]	NA	368	3688	1.26 (1.11-1.44)	<.001	1.26 (1.11-1.44)	<.001
Some college or technical degree	1565	13 850	1.36 (1.27-1.45)	<.001	198	1666	1.55 (1.31-1.83)	<.001	1.14 (0.96-1.35)	.13
≤High school	770	6129	1.54 (1.41-1.69)	<.001	60	405	1.92 (1.45-2.55)	<.001	1.25 (0.93-1.66)	.13
**Black/African American** ^c^										
≥College	21	368	1 [Reference]	NA	183	1738	1.94 (1.21-3.10)	.01	1.94 (1.21-3.10)	.01
Some college or technical degree	30	222	2.38 (1.32-4.28)	<.001	76	803	1.72 (1.04-2.85)	.03	0.72 (0.46-1.14)	.17
≤High school	8	114	1.28 (0.55-2.99)	.57	20	135	2.99 (1.56-5.75)	<.001	2.34 (0.98-5.60)	.06
**Latina** ^d^										
≥College	47	560	1 [Reference]	NA	29	407	0.90 (0.55-1.48)	.69	0.90 (0.55-1.48)	.21
Some college or technical degree	61	470	1.61 (1.07-2.42)	.02	26	196	1.82 (1.09-3.06)	.02	1.13 (0.69-1.87)	.63
≤High school	32	279	1.50 (0.92-2.45)	.10	5	81	0.80 (0.31-2.10)	.65	0.53 (0.20-1.42)	.69
**Non-Hispanic White** ^e^										
≥College	2150	25 117	1 [Reference]	NA	156	1543	1.20 (1.01-1.42)	.04	1.20 (1.01-1.42)	.04
Some college or technical degree	1474	13 158	1.34 (1.25-1.44)	<.001	96	667	1.76 (1.42-2.20)	<.001	1.32 (1.05-1.64)	.02
≤High school	730	5736	1.56 (1.43-1.71)	<.001	35	189	2.24 (1.56-3.23)	<.001	1.43 (0.99-2.08)	.06

Abbreviations: NA, not applicable; OR, odds ratio.

^a^ Conditional logistic regression models are adjusted for age in 5-year increments, race/ethnicity (in the overall population; Black or African American [non-Hispanic], Latina, or non-Hispanic White), longest lived region of residence (Northeast, Midwest, South, West, Puerto Rico, or outside the US and Puerto Rico), and current employment (yes or no). Cases and controls were race and ethnicity– and age-matched.

^b^ Measures of effect modification on the additive scale in the overall population: relative excess risk due to interaction (RERI) for some college vs college or higher, −0.07 (95% CI, −0.35 to 0.21); RERI for high school or less vs college or higher, 0.11 (95% CI, −0.44 to 0.67). Measures of effect modification on the multiplicative scale in the overall population: ratio of ORs for some college vs college or higher, 0.90 (95% CI, 0.74 to 1.10); ratio of ORs for high school or less vs college or higher, 0.99 (95% CI, 0.73 to 1.34).

^c^ Measures of effect modification on the additive scale among Black or African American women: RERI for some college vs college or higher, −1.60 (95% CI, −3.15 to −0.04); RERI for high school or less vs college or higher, 0.77 (95% CI, −0.97 to 2.52). Measures of effect modification on the multiplicative scale among BAA women: ratio of ORs for some college vs college or higher, 0.37 (95% CI, 0.19 to 0.72); ratio of ORs for high school or less vs college or higher, 1.21 (95% CI, 0.45 to 3.25).

^d^ Measures of effect modification on the additive scale among Latina women: RERI for some college vs college or higher, 0.31 (95% CI, −0.66 to 1.28); RERI for high school or less vs college or higher, −0.61 (95% CI, −1.71 to 0.50). Measures of effect modification on the multiplicative scale among Latina women: ratio of ORs for some college vs college or higher, 1.25 (95% CI, 0.62 to 2.52); ratio of ORs for high school or less vs college or higher, 0.59 (95% CI, 0.20 to 1.77).

^e^ Measures of effect modification on the additive scale among non-Hispanic White women: RERI for some college vs college or higher, 0.23 (95% CI, −0.21 to 0.66); RERI for high school or less vs college or higher, 0.48 (95% CI, −0.36 to 1.32). Measures of effect modification on the multiplicative scale among non-Hispanic White women: ratio of ORs for some college vs college or higher, 1.10 (95% CI, 0.83 to 1.45); ratio of ORs for high school or less vs college or higher, 1.20 (95% CI, 0.80 to 1.80).

### Sensitivity Analyses

After further adjustment for potential mediators, including additional sociodemographic characteristics and health behaviors, results, although slightly attenuated, were consistent with the main analysis (eTables 6-8 in Supplement 1). Associations between both perceived everyday and either everyday or major RED remained stronger among Black women with college or higher education vs some college. Further adjustment for birthplace among Latina women yielded similar results to the main analysis, with the suggestion of stronger associations with perceived major RED among women reporting some college compared with college or higher education (eTable 9 in Supplement 1).

## Discussion

In this nested case-control study of White, Black, and Latina middle-to-older aged US women, educational attainment did not modify associations with major RED but did modify the association between everyday RED and higher risk of hypertension solely among Black women. Specifically, everyday RED–associated hypertension risk was higher among Black women who completed college or higher than among Black women who completed some college. Everyday RED–associated hypertension risk was comparable between Black women with college or higher education and with high school or less education. Results suggest a possible U-shaped relationship by educational attainment. Black women who attained college or higher education had the highest prevalence of perceived everyday and major RED. Furthermore, hypertension incidence was highest among Black women irrespective of educational attainment. These findings—consistent with our hypotheses and intersectionality frameworks^8,9^—not only reiterate the high burden of hypertension among Black women but also importantly demonstrate notable within-group differences likely related to elevated racism-related stressors and limited buffering by educational attainment, resulting in exacerbated hypertension risk among Black women achieving the highest levels of education.^20^ This highlights the need for tailored interventions designed to reduce the burden of hypertension among Black women.^44^

Our novel results highlighting educational attainment as a potential modifier of RED-hypertension within Black women are plausible when considering prior discrimination literature.^23^ Prior studies have demonstrated links between RED and hypertension across multiethnic populations and varying age ranges.^6,18,25,26,30,31,45^ Studies have also shown that health benefits related to higher educational attainment are not equitable among Black and Latina compared to White adults, which is likely related to the pervasiveness of structural racism and other forms of discrimination faced by minoritized racial and ethnic groups.^9,19,20,21,22,23,46,47^ Our findings of EM by educational attainment for everyday RED but not major RED add valuable insights into RED’s health impacts. Highly educated Black women may encounter everyday discriminatory acts, such as microaggressions and unfair treatment, more frequently due to residing, working, and participating in environments where minoritized groups were historically excluded and are currently sometimes unwelcomed.^9,20^ The chronic nature of the exposure may contribute to exacerbated risk of hypertension associated with experiencing RED by contributing to daily stress, vigilance, denial of resources despite perceived increased access, loneliness, isolation, and internalized racism.^7,12^ Chronic RED can activate stress response pathways, contribute to insalubrious coping behaviors, disrupt sleep, and contribute to physiological wear-and-tear, all of which are associated with higher burden of hypertension.^11,12,13,14^

Counter to our hypothesis, educational attainment did not modify associations between RED and higher hypertension risk among Latina women. However, this may be related to our assessment not adequately capturing the complexity of colorism or skin color discrimination that has previously been associated with health and well-being among Latino populations.^48^ Moreover, approximately one-third of Latina women in our sample resided in Puerto Rico. Limited geographic and ethnic diversity may have impacted our results. Future studies with culturally relevant discrimination assessments among diverse Latina populations with varying heritage and skin color phenotypes are warranted. Nonetheless, RED appears associated with hypertension among Black, Latina, and White women and can serve as a modifiable target for hypertension reduction among US women.

### Limitations and Strengths

This study has limitations. All data were self-reported and therefore subject to misclassification. However, appraisal of RED as a stressor whether, in fact, actual or perceived can cause stress and has health implications.^49^ Furthermore, self-reported hypertension has been validated as a useful measure in the absence of objective measures among White and Black US women.^50^ RED was measured in broad categories, as ever vs never, and did not include other important forms of RED (eg, physically attacked), thus potentially missing important nuances related to discrimination, such as perceived severity, frequency, timing, and duration, that may modify the impact of RED on health.^45,51^ Residual confounding is also possible; however, our results remained consistent after adjustment for robust sets of both potential confounders and potential mediators. Exclusions could have introduced selection bias, as there were differences between included and excluded participants. Included participants were predominately White, had higher socioeconomic status, and had better health profiles. However, RED did not differ between included and excluded participants. Smaller sample sizes of minoritized racial and ethnic groups and stratification resulted in limited power to detect associations and EM. Results are likely an underestimation of associations between RED and hypertension. The Sister Study population largely has higher socioeconomic status and is mostly White and middle-to-older aged, thus limiting generalizability. Replication, including studies using life course frameworks and objective measures of hypertension among samples including more racially and ethnically as well as socioeconomically diverse populations, of other genders, and across age groups are warranted.

Despite these limitations, notable strengths include our nested case-control design within a large national prospective cohort, increasing power and reducing immortal time bias related to the earlier hypertension onset among Black women. Strengths also include use of causal inference techniques to guide adjustment sets, the diversity in race and ethnicity and educational attainment in the sample, and our ability to stratify by both potential modifiers.

## Conclusions

In this study, Black women who attained a Bachelor’s degree or higher were the most likely to report RED and, compared with Black women with some college, had stronger associations between everyday RED and higher hypertension risk. RED was also associated with higher hypertension risk among White and Latina women. Mitigation of RED appears beneficial for all. Assessment of experiences of discrimination and educational attainment may inform hypertension prevention, management, and intervention efforts implemented by health professionals. Importantly, given the highest burdens of both RED and hypertension incidence among Black women and because educational attainment appeared as an amplifier rather than a buffer, multilevel interventions across policy, workplace, health care, community, and interpersonal settings are warranted to address racism, racism-related stress, and disproportionate burdens of hypertension.

## Notes

Supplement 1. **eFigure 1.** Flow Chart of Incidence Density Sampling Pool Creation **eFigure 2.** Directed Acyclic Graph **eTable 1.** Comparison of Ineligible Non-Hispanic White, Non-Hispanic Black or African American, and Hispanic or Latina Participants Without Prior Hypertension Diagnosis to Eligible Controls, Sister Study **eTable 2.** Case and Control Characteristics Among Non-Hispanic Black or African American Women, Overall and by Educational Attainment, Sister Study **eTable 3.** Case and Control Characteristics Among Hispanic or Latina Women, Overall and by Educational Attainment, Sister Study **eTable 4.** Case and Control Characteristics Among Non-Hispanic White Women, Overall and by Educational Attainment, Sister Study **eTable 5.** Results of Conditional Logistic Regression: Odds Ratios for Associations Between Reports of Racial and Ethnic Discrimination and Hypertension Incidence, Overall and Stratified by Race and Ethnicity, Sister Study, 2003-2019 **eTable 6.** Results of Conditional Logistic Regression With Additional Adjustment for Potential Mediators: Odds Ratios for Associations Between Reports of Everyday Racial and Ethnic Discrimination and Hypertension Incidence Overall and Within Racial and Ethnic Groups, Stratified by Educational Attainment, Sister Study, 2003-2019 **eTable 7.** Results of Conditional Logistic Regression With Additional Adjustment for Potential Mediators: Odds Ratios for Associations Between Reports of Major Racial and Ethnic Discrimination and Hypertension Incidence Overall and Within Racial and Ethnic Groups, Stratified by Educational Attainment, Sister Study, 2003-2019 **eTable 8.** Results of Conditional Logistic Regression With Additional Adjustment for Potential Mediators: Odds Ratios for Associations Between Reports of Either Everyday or Major Racial and Ethnic Discrimination and Hypertension Incidence Overall and Within Racial and Ethnic Groups, Stratified by Educational Attainment, Sister Study, 2003-2019 **eTable 9.** Results of Conditional Logistic Regression With Additional Adjustment for Birthplace: Odds Ratios for Associations Between Reports of Everyday, Major, and Either Everyday or Major Racial and Ethnic Discrimination and Hypertension Incidence Among Latina Women, Stratified by Educational Attainment, Sister Study, 2003-2019

Supplement 2.
Data Sharing Statement
