# Supplementary material for: Racial and Ethnic Discrimination and Hypertension by Educational Attainment Among a Cohort of US Women
Source: JAMA Netw Open. 2023 Nov 22;6(11):e2344707. doi: 10.1001/jamanetworkopen.2023.44707 (PMC10665977; doi:10.1001/jamanetworkopen.2023.44707)
Supplement: Supplement 1. — eFigure 1. Flow Chart of Incidence Density Sampling Pool Creation eFigure 2. Directed Acyclic Graph eTable 1. Comparison of Ineligible Non-Hispanic White, Non-Hispanic Black or African American, and Hispanic or Latina Participants Without Prior Hypertension Diagnosis to Eligible Controls, Sister Study eTable 2. Case and Control Characteristics Among Non-Hispanic Black or African American Women, Overall and by Educational Attainment, Sister Study eTable 3. Case and Control Characteristics Among Hispanic or Latina Women, Overall and by Educational Attainment, Sister Study eTable 4. Case and Control Characteristics Among Non-Hispanic White Women, Overall and by Educational Attainment, Sister Study eTable 5. Results of Conditional Logistic Regression: Odds Ratios for Associations Between Reports of Racial and Ethnic Discrimination and Hypertension Incidence, Overall and Stratified by Race and Ethnicity, Sister Study, 2003-2019 eTable 6. Results of Conditional Logistic Regression With Additional Adjustment for Potential Mediators: Odds Ratios for Associations Between Reports of Everyday Racial and Ethnic Discrimination and Hypertension Incidence Overall and Within Racial and Ethnic Groups, Stratified by Educational Attainment, Sister Study, 2003-2019 eTable 7. Results of Conditional Logistic Regression With Additional Adjustment for Potential Mediators: Odds Ratios for Associations Between Reports of Major Racial and Ethnic Discrimination and Hypertension Incidence Overall and Within Racial and Ethnic Groups, Stratified by Educational Attainment, Sister Study, 2003-2019 eTable 8. Results of Conditional Logistic Regression With Additional Adjustment for Potential Mediators: Odds Ratios for Associations Between Reports of Either Everyday or Major Racial and Ethnic Discrimination and Hypertension Incidence Overall and Within Racial and Ethnic Groups, Stratified by Educational Attainment, Sister Study, 2003-2019 eTable 9. Results of Conditional Logistic Regression With Addit [file jamanetwopen-e2344707-s001.pdf]

Supplemental Online Content

Gaston SA, Forde AT, Green M, Sandler DP, Jackson CL. Racial and ethnic discrimination and hypertension by educational attainment among a cohort of US women. *JAMA Netw Open.* 2023;6(11):e2344707. doi:10.1001/jamanetworkopen.2023.44707

eFigure 1. Flow Chart of Incidence Density Sampling Pool Creation

eFigure 2. Directed Acyclic Graph

eTable 1. Comparison of Ineligible Non-Hispanic White, Non-Hispanic Black or African American, and Hispanic or Latina Participants Without Prior Hypertension Diagnosis to Eligible Controls, Sister Study

eTable 2. Case and Control Characteristics Among Non-Hispanic Black or African American Women, Overall and by Educational Attainment, Sister Study

eTable 3. Case and Control Characteristics Among Hispanic or Latina Women, Overall and by Educational Attainment, Sister Study

eTable 4. Case and Control Characteristics Among Non-Hispanic White Women, Overall and by Educational Attainment, Sister Study

eTable 5. Results of Conditional Logistic Regression: Odds Ratios for Associations Between Reports of Racial and Ethnic Discrimination and Hypertension Incidence, Overall and Stratified by Race and Ethnicity, Sister Study, 2003-2019

eTable 6. Results of Conditional Logistic Regression With Additional Adjustment for Potential Mediators: Odds Ratios for Associations Between Reports of Everyday Racial and Ethnic Discrimination and Hypertension Incidence Overall and Within Racial and Ethnic Groups, Stratified by Educational Attainment, Sister Study, 2003-2019

eTable 7. Results of Conditional Logistic Regression With Additional Adjustment for Potential Mediators: Odds Ratios for Associations Between Reports of Major Racial and Ethnic Discrimination and Hypertension Incidence Overall and Within Racial and Ethnic Groups, Stratified by Educational Attainment, Sister Study, 2003-2019

eTable 8. Results of Conditional Logistic Regression With Additional Adjustment for Potential Mediators: Odds Ratios for Associations Between Reports of Either Everyday or Major Racial and Ethnic Discrimination and Hypertension Incidence Overall and Within Racial and Ethnic Groups, Stratified by Educational Attainment, Sister Study, 2003-2019

eTable 9. Results of Conditional Logistic Regression With Additional Adjustment for Birthplace: Odds Ratios for Associations Between Reports of Everyday, Major, and

© 2023 Gaston SA et al. *JAMA Network Open.*

Either Everyday or Major Racial and Ethnic Discrimination and Hypertension Incidence Among Latina Women, Stratified by Educational Attainment, Sister Study, 2003-2019

This supplemental material has been provided by the authors to give readers additional information about their work.

© 2023 Gaston SA et al. *JAMA Network Open.*

eFigure 1. Flow Chart of Incidence Density Sampling Pool Creation

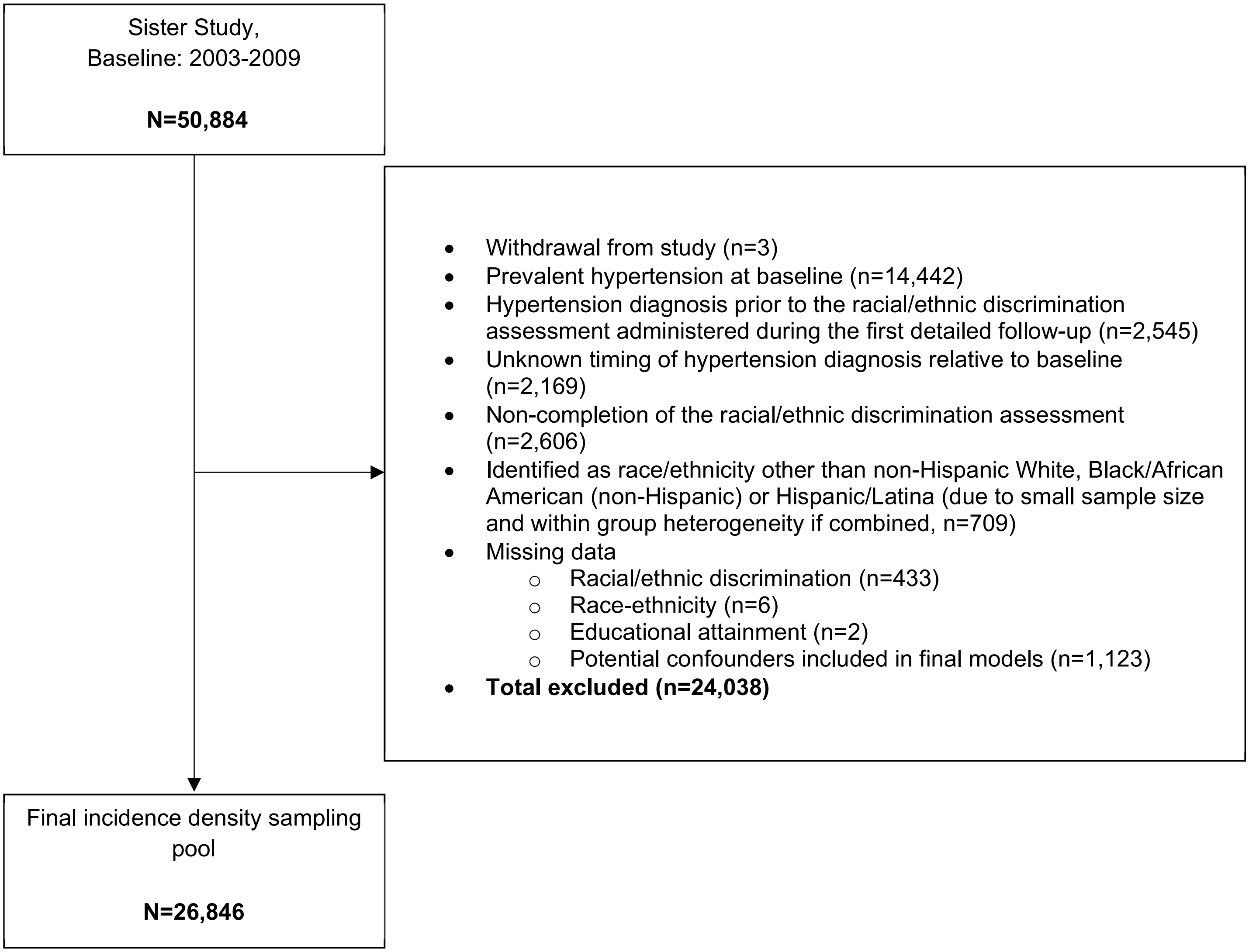

© 2023 Gaston SA et al. *JAMA Network Open.*

eFigure 2. Directed Acyclic Graph

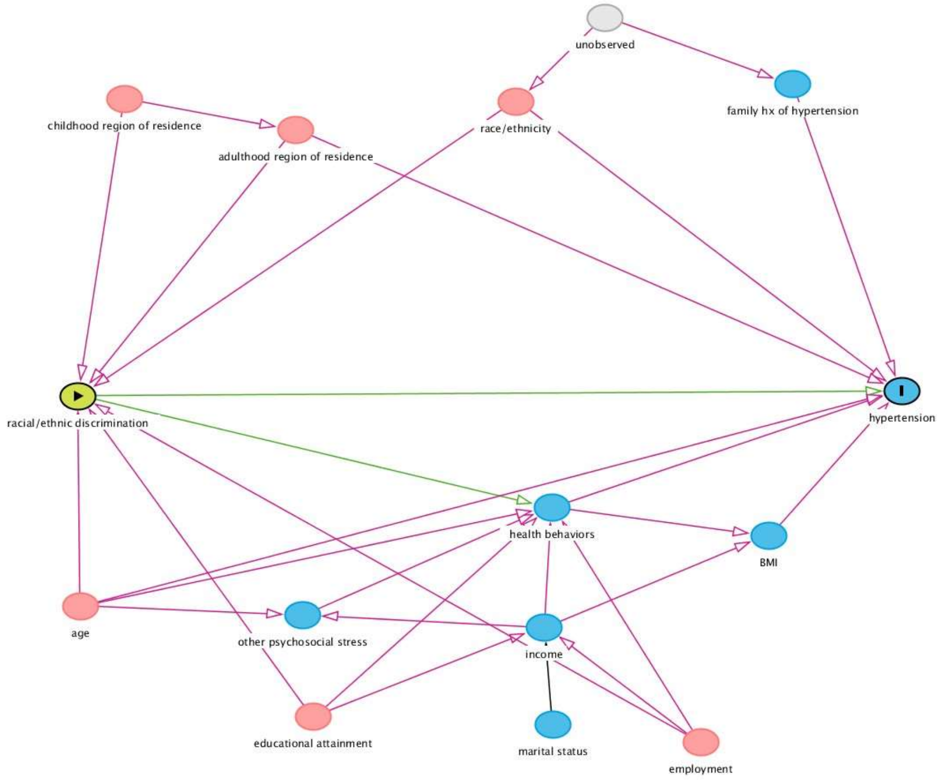

© 2023 Gaston SA et al. *JAMA Network Open.*

eTable 1. Comparison of Ineligible Non-Hispanic White, Non-Hispanic Black or African American, and Hispanic or Latina Participants Without Prior Hypertension Diagnosis to Eligible Controls, Sister Study

	Eligible			Ineligible		
**N or n (%)**	**26,846**			**6,185**		
**Developed hypertension over follow-up, n**	**5,197**			**718**		
	**Mean or n**	**±SD or %**	**% missing**	**Mean or n**	**±SD or %**	**% missing**
**Sociodemographic Characteristics**						
Mean age ± SD, years *	53.4	± 8.7		54.2	± 9.3	
Age Category *						
≤55 years	16,040	59.7		3,457	55.9	
>55 years	10,806	40.3		2,728	44.1	
Birth Cohort *						
Silent Generation (1928-1945)	5,955	22.2		1,646	26.6	
Baby Boomer (1946-1964)	18,582	69.2		3,950	63.9	
Generation X (1965-1980)	2,309	8.6		586	9.5	
Race/ethnicity *						
non-Hispanic White	24,370	90.8		4,961	80.2	
non-Hispanic Black/African American	1,335	5.0		763	12.3	
Hispanic/Latina	1,141	4.3		461	7.5	
Region of residence lived longest as an adult *						0.4
Northeast	5,270	19.6		1,148	18.6	
Midwest	7,716	28.7		1,591	25.7	
South	7,538	28.1		1,875	30.3	
West	5,841	21.8		1,323	21.4	
Puerto Rico or outside US and Puerto Rico	481	1.8		218	3.5	
Marital Status *			<0.01			0.1
Married/living as married	21,164	78.8		4,366	70.6	
Single/never married	1,331	5.0		412	6.7	
Divorced/separated/widowed	4,349	16.2		1,403	22.7	
Educational attainment *						0.1
≤ High school	3,485	13.0		951	15.4	
Some college or technical degree	8,272	30.8		2,248	36.3	
≥ College (Bachelor's or greater)	15,089	56.2		2,981	48.2	
Currently employed (yes) *	18,897	70.4		3,831	61.9	5.3
Annual household income *						
Less than $20,000	777	2.9		368	5.9	
$20,000 to $49,999	4,575	17.0		1,410	22.8	
$50,000 to $99,999	11,022	41.1		2,466	39.9	
$100,000 or more	10,472	39.0		1,938	31.3	
**Health Behaviors**						
Smoking Status *						0.1

© 2023 Gaston SA et al. *JAMA Network Open.*

	Eligible			Ineligible		
**N or n (%)**	**26,846**			**6,185**		
**Developed hypertension over follow-up, n**	**5,197**			**718**		
	**Mean or n**	**±SD or %**	**% missing**	**Mean or n**	**±SD or %**	**% missing**
Current	1,936	7.2		676	10.9	
Former	9,306	34.7		2,164	35.0	
Never	15,604	58.1		3,337	54.0	
Alcohol Consumption (past 12 months) *						0.8
Current, ≥2 drinks/day	1,269	4.7		307	5.0	
Current, <1 to <2 drinks/day	21,470	80.0		4,587	74.2	
Never/Former	4,107	15.3		1,244	20.1	
Log-METs-hours/week ± SD *	3.8	± 0.6		3.7	± 0.7	3.9
Healthy Eating Index score ± SD *	72.3	± 9.5		71.3	± 9.9	11.0
Mean sleep score ± SD *	1.0	± 1.2		1.2	± 1.3	4.7
**Clinical Characteristics**						
BMI category *			<0.01			0.2
Underweight (<18.5 kg/m^2)	399	1.5		76	1.2	
Recommended weight (18.5-24.9 kg/m^2)	12,818	47.7		2,134	34.5	
Overweight (25.0-29.9 kg/m^2)	8,429	31.4		1,999	32.3	
Obesity (≥30.0 kg/m^2)	5,193	19.3		1,966	31.8	
Postmenopausal (yes) *	15,949	59.4		3,868	62.5	0.2

Data presented as % or mean ± SD. Percentages may not sum to 100 due to missing values and rounding.

Abbreviations: SD (standard deviation); US (United States); METs (metabolic equivalent of tasks); BMI (body mass index)
Note: Proportion employed is calculated as: number employed/(number employed + unemployed + homemaker + student + retired). Healthy Eating Index scores range from 0-100 with higher scores indicating a healthier diet. Sleep score is a summary score for 6 poor sleep dimensions. Sleep score ranges from 0-6. Participants were assigned a value of one for each if they reported experiencing the following: (1) habitual short [<7-hours] or long [>9-hours] sleep duration (vs. recommended [7-9-hours]); (2) inconsistent weekly sleep patterns, defined as inconsistent wake-up times and bedtimes during the prior six weeks (yes vs. no); (3) sleep debt, defined as ≥2-hour difference between average longest and shortest sleep duration (yes vs. no); (4) frequent napping (≥3 days/week versus <3 days/week); (5) difficulty falling asleep, defined as taking >30 minutes vs. ≤ 30 minutes to fall asleep on average; and (6) difficulty staying asleep, defined as waking up ≥3 times per night ≥3 nights/week vs. <3 times per night <3 nights/week.

* Chi-Square or unpaired samples t-test two-sided p-value <0.05.

© 2023 Gaston SA et al. *JAMA Network Open.*

eTable 2. Case and Control Characteristics Among Non-Hispanic Black or African American Women, Overall and by Educational Attainment, Sister Study

	Total				≥ College				Some College				≤ High School			
	Hypertension Cases		Controls		Hypertension Cases		Controls		Hypertension Cases		Controls		Hypertension Cases		Controls	
**N or n (%)**	**338**		**3,380**		**204**		**2,106**		**106**		**1,025**		**28**		**249**	
	**mean**	**±SD or %**	**mean**	**±SD or %**	**mean**	**±SD or %**	**mean**	**±SD or %**	**mean**	**±SD or %**	**mean**	**±SD or %**	**mean**	**±SD or %**	**mean**	**±SD or %**
**Sociodemographic Characteristics**																
Mean age ± SD, years	51.0	± 7.9	52.2	± 7.8	51.4	± 8.	52.0	± 7.6	50.1	± 7.9	52.4	± 7.9	51.8	± 7.2	52.8	± 8.2
Age Category																
≤55 years		72.8		65.9		70.1		66.7		77.4		64.3		75.0		65.1
>55 years		27.2		34.1		29.9		33.3		22.6		35.7		25.0		34.9
Region of residence lived longest as an adult																
Northeast		12.1		13.6		10.8		13.2		9.4		12.8		32.1		19.7
Midwest		23.4		23.7		22.5		24.1		24.5		23.5		25.0		20.9
South		57.1		51.6		57.4		51.0		61.3		52.7		39.3		52.2
West		6.8		10.2		8.3		10.8		4.7		10.1		3.6		5.6
Puerto Rico or outside US and Puerto Rico		0.6		0.9		1.0		0.9		0.0		0.9		0.0		1.6
Marital Status																
Married/living as married		54.1		58.2		52.9		58.9		60.4		58.2		39.3		51.8
Single/never married		14.5		12.7		14.2		13.6		16.0		11.6		10.7		10.0
Divorced/separated/widowed		31.4		29.0		32.8		27.5		23.6		29.9		50.0		38.2
Educational attainment																
≤ High school		8.3		7.4		0.0		0.0		0.0		0.0		100.0		100.0
Some college or technical degree		31.4		30.3		0.0		0.0		100.0		100.0		0.0		0.0
≥ College (Bachelor's or greater)		60.4		62.3		100.0		100.0		0.0		0.0		0.0		0.0
Currently employed (yes)		78.7		80.4		80.4		84.4		77.4		74.3		71.4		71.9
Annual household income																
Less than $20,000		2.7		4.5		0.5		1.4		4.7		7.9		10.7		16.5
$20,000 to $49,999		23.4		21.5		15.7		14.9		30.2		30.1		53.6		41.8
$50,000 to $99,999		44.1		41.6		45.6		43.4		44.3		40.4		32.1		30.9

© 2023 Gaston SA et al. *JAMA Network Open.*

	Total				≥ College				Some College				≤ High School			
	Hypertension Cases		Controls		Hypertension Cases		Controls		Hypertension Cases		Controls		Hypertension Cases		Controls	
**N or n (%)**	**338**		**3,380**		**204**		**2,106**		**106**		**1,025**		**28**		**249**	
	**mean**	**±SD or %**	**mean**	**±SD or %**	**mean**	**±SD or %**	**mean**	**±SD or %**	**mean**	**±SD or %**	**mean**	**±SD or %**	**mean**	**±SD or %**	**mean**	**±SD or %**
$100,000 or more		29.9		32.4		38.2		40.2		20.8		21.6		3.6		10.8
**Health Behaviors**																
Smoking Status																
Current		10.1		7.4		8.3		5.4		8.5		8.4		28.6		19.7
Former		22.8		23.8		20.6		22.1		24.5		26.7		32.1		26.1
Never		67.2		68.8		71.1		72.5		67.0		64.9		39.3		54.2
Alcohol Consumption (past 12 months)																
Current, ≥2 drinks/day		1.2		0.8		0.5		0.4		0.9		1.7		7.1		0.4
Current, <1 to <2 drinks/day		72.2		72.3		72.1		75.1		73.6		68.3		67.9		64.7
Never/Former		26.6		27.0		27.5		24.5		25.5		30.0		25.0		34.9
Log-METs-hours/week ± SD	3.6	± 0.7	3.7	± 0.7	3.6	± 0.7	3.6	± 0.7	3.7	± 0.7	3.7	± 0.6	3.6	± 0.8	3.6	± 0.7
Healthy Eating Index score ± SD	70.9	± 9.8	72.0	± 9.1	72.3	± 9.6	72.9	± 8.7	69.1	± 9.7	70.9	± 9.5	67.1	± 9.5	69.1	± 10.0
Mean sleep score ± SD	1.6	± 1.3	1.5	± 1.3	1.5	± 1.2	1.3	± 1.2	1.8	± 1.4	1.6	± 1.3	1.9	± 1.5	2.3	± 1.6
**Clinical Characteristics**																
BMI category																
Underweight (<18.5 kg/m^2)		0.6		0.8		0.5		0.7		0.9		1.2		0.0		0.4
Recommended weight (18.5-24.9 kg/m^2)		19.5		28.1		23.5		30.3		14.2		24.9		10.7		22.9
Overweight (25.0-29.9 kg/m^2)		33.7		36.5		32.4		38.3		35.8		32.3		35.7		39.0
Obesity (≥30.0 kg/m^2)		46.2		34.6		43.6		30.7		49.1		41.7		53.6		37.8
Postmenopausal (yes)		52.7		55.3		51.0		52.6		53.8		58.1		60.7		66.7
**Racial/ethnic Discrimination**																
Everyday racial/ethnic discrimination																
Yes		76.3		74.0		83.3		77.0		66.0		73.5		64.3		51.4
No		23.7		26.0		16.7		23.0		34.0		26.5		35.7		48.6
Major racial/ethnic discrimination																

© 2023 Gaston SA et al. *JAMA Network Open.*

	Total				≥ College				Some College				≤ High School			
	Hypertension Cases		Controls		Hypertension Cases		Controls		Hypertension Cases		Controls		Hypertension Cases		Controls	
**N or n (%)**	**338**		**3,380**		**204**		**2,106**		**106**		**1,025**		**28**		**249**	
	**mean**	**±SD or %**	**mean**	**±SD or %**	**mean**	**±SD or %**	**mean**	**±SD or %**	**mean**	**±SD or %**	**mean**	**±SD or %**	**mean**	**±SD or %**	**mean**	**±SD or %**
Yes		54.1		51.0		59.3		55.6		44.3		46.0		53.6		67.1
No		45.9		49.0		40.7		44.4		55.7		54.0		46.4		32.9
Either everyday or major racial/ethnic discrimination																
Yes		82.5		79.2		89.8		82.5		71.7		78.3		71.4		54.2
No		17.5		20.8		10.3		17.5		28.3		21.7		28.6		45.8

Data presented as % or mean ± SD. Percentages may not sum to 100 due to missing values and rounding.

Abbreviations: SD (standard deviation); US (United States); METs (metabolic equivalent of tasks); BMI (body mass index)

Note: Proportion employed is calculated as: number employed/(number employed + unemployed + homemaker + student + retired). Healthy Eating Index scores range from 0-100 with higher scores indicating a healthier diet. Sleep score is a summary score for 6 poor sleep dimensions. Sleep score ranges from 0-6. Participants were assigned a value of one for each if they reported experiencing the following: (1) habitual short [<7-hours] or long [>9-hours] sleep duration (vs. recommended [7-9-hours]); (2) inconsistent weekly sleep patterns, defined as inconsistent wake-up times and bedtimes during the prior six weeks (yes vs. no); (3) sleep debt, defined as ≥2-hour difference between average longest and shortest sleep duration; (4) frequent napping (≥3 days/week versus <3 days/week); (5) difficulty falling asleep, defined as taking >30 minutes vs. ≤ 30 minutes to fall asleep on average; and (6) difficulty staying asleep, defined as waking up ≥3 times per night ≥3 nights/week vs. <3 times per night <3 nights/week.

Missingness: <0.01% for marital status and BMI category

© 2023 Gaston SA et al. *JAMA Network Open.*

eTable 3. Case and Control Characteristics Among Hispanic or Latina Women, Overall and by Educational Attainment, Sister Study

	Total				≥ College				Some College				≤ High School			
	Hypertension Cases		Controls		Hypertension Cases		Controls		Hypertension Cases		Controls		Hypertension Cases		Controls	
N or n	200		1,993		76		967		87		666		37		360	
	mean	±SD or %	mean	±SD or %	mean	±SD or %	mean	±SD or %	mean	±SD or %	mean	±SD or %	mean	±SD or %	mean	±SD or %
**Sociodemographic Characteristics**																
Mean age ± SD, years	52.6	± 8.7	53.1	± 8.5	51.8	± 9.0	52.1	± 8.4	52.3	± 8.3	53.6	± 8.4	55.0	± 9.1	54.9	± 8.4
Age Category																
≤55 years		63.0		62.9		61.8		67.9		66.7		61.9		56.8		51.1
>55 years		37.0		37.1		38.2		32.1		33.3		38.1		43.2		48.9
Region of residence lived longest as an adult																
Northeast		10.0		8.8		10.5		9.4		9.2		8.3		10.8		8.1
Midwest		8.0		6.9		6.6		7.5		8.0		4.5		10.8		9.4
South		20.5		25.6		25.0		26.6		18.4		26.9		16.2		20.8
West		23.0		27.4		15.8		24.2		29.9		30.2		21.6		31.1
Puerto Rico or outside US and Puerto Rico		38.5		31.3		42.1		32.3		34.5		30.2		40.5		30.6
Marital Status																
Married/living as married		66.0		71.6		67.1		71.0		60.9		71.6		75.7		73.1
Single/never married		5.0		5.8		9.2		6.5		2.3		5.7		2.7		4.2
Divorced/separated/widowed		29.0		22.6		23.7		22.4		36.8		22.7		21.6		22.8
Educational attainment																
≤ High school		18.5		18.1		0.0		0.0		0.0		0.0		100.0		100.0
Some college or technical degree		43.5		33.4		0.0		0.0		100.0		100.0		0.0		0.0
≥ College (Bachelor's or greater)		38.0		48.5		100.0		100.0		0.0		0.0		0.0		0.0
Currently employed (yes)		66.0		64.9		69.7		70.9		69.0		64.3		51.4		50.0

© 2023 Gaston SA et al. *JAMA Network Open.*

Annual household income								
Less than $20,000	20.0	12.1	10.5	6.2	19.5	12.5	40.5	27.2
$20,000 to $49,999	29.0	25.5	28.9	17.8	27.6	30.8	32.4	36.4
$50,000 to $99,999	33.0	36.8	31.6	39.2	39.1	38.1	21.6	28.1
$100,000 or more	18.0	25.6	28.9	36.8	13.8	18.6	5.4	8.3
**Health Behaviors**								
Smoking Status								
Current	6.0	5.8	6.6	3.9	3.4	2.7	10.8	7.8
Former	29.5	24.7	22.4	22.6	67.8	74.2	43.2	23.6
Never	64.5	69.5	71.1	73.4	28.7	23.1	45.9	68.6
Alcohol Consumption (past 12 months)								
Current, ≥2 drinks/day	1.5	1.7	0.0	0.5	3.4	2.7	0.0	2.8
Current, <1 to <2 drinks/day	68.5	74.8	76.3	79.8	67.8	74.2	54.1	62.2
Never/Former	30.0	23.6	23.7	19.6	28.7	23.1	45.9	35.0
Log-METs-hours/week ± SD	3.7 ± 0.7	3.8 ± 0.7	3.7 ± 0.6	3.8 ± 0.6	3.7 ± 0.7	3.7 ± 0.7	3.7 ± 0.7	3.8 ± 0.7
Healthy Eating Index score ± SD	70.4 ± 9.8	71.1 ± 9.9	71.5 ± 8.9	72.3 ± 9.8	70.2 ± 10.4	70.1 ± 9.9	68.5 ± 10.3	69.9 ± 9.9
Mean sleep score ± SD	1.4 ± 1.4	1.3 ± 1.3	1.1 ± 1.3	1.2 ± 1.2	1.6 ± 1.5	1.4 ± 1.4	1.8 ± 1.2	1.6 ± 1.5
**Clinical Characteristics**								
BMI category								
Underweight (<18.5 kg/m^2)	1.0	0.7	1.3	0.6	1.1	0.9	0.0	0.3
Recommended weight (18.5-24.9 kg/m^2)	25.5	40.5	28.9	46.8	26.4	36.9	16.2	30.3
Overweight (25.0-29.9 kg/m^2)	44.5	37.6	42.1	35.0	41.4	36.5	56.8	46.9
Obesity (≥30.0 kg/m^2)	29.0	21.2	27.6	17.6	31.0	25.7	27.0	22.5

© 2023 Gaston SA et al. *JAMA Network Open.*

Postmenopausal (yes)	61.0	59.5	53.9	54.1	58.6	59.8	81.1	73.3
**Racial/ethnic Discrimination**								
Everyday racial/ethnic discrimination								
Yes	27.0	31.2	36.8	39.2	26.4	26.7	8.1	17.8
No	73.0	68.8	63.2	60.8	73.6	73.3	91.9	82.2
Major racial/ethnic discrimination								
Yes	14.5	13.9	17.1	18.8	16.1	9.3	5.4	9.4
No	85.5	86.1	82.9	81.2	83.9	90.7	94.6	90.6
Either everyday or major racial/ethnic discrimination								
Yes	30.0	34.3	38.2	42.1	29.9	29.4	13.5	22.5
No	70.0	65.7	61.8	57.9	70.1	70.6	86.5	77.5

Data presented as % or mean ± SD. Percentages may not sum to 100 due to missing values and rounding.

Abbreviations: SD (standard deviation); US (United States); METs (metabolic equivalent of tasks); BMI (body mass index)

Note: Proportion employed is calculated as: number employed/(number employed + unemployed + homemaker + student + retired). Healthy Eating Index scores range from 0-100 with higher scores indicating a healthier diet. Sleep score is a summary score for 6 poor sleep dimensions. Sleep score ranges from 0-6. Participants were assigned a value of one for each if they reported experiencing the following: (1) habitual short [<7-hours] or long [>9-hours] sleep duration (vs. recommended [7-9-hours]); (2) inconsistent weekly sleep patterns, defined as inconsistent wake-up times and bedtimes during the prior six weeks (yes vs. no); (3) sleep debt, defined as ≥2-hour difference between average longest and shortest sleep duration (yes vs. no); (4) frequent napping (≥3 days/week versus <3 days/week); (5) difficulty falling asleep, defined as taking >30 minutes vs. ≤ 30 minutes to fall asleep on average; and (6) difficulty staying asleep, defined as waking up ≥3 times per night ≥3 nights/week vs. <3 times per night <3 nights/week.

Missingness: <0.01% for marital status and BMI category

© 2023 Gaston SA et al. *JAMA Network Open.*

eTable 4. Case and Control Characteristics Among Non-Hispanic White Women, Overall and by Educational Attainment, Sister Study

	Total				≥ College				Some College				≤ High School			
	Hypertension Cases		Controls		Hypertension Cases		Controls		Hypertension Cases		Controls		Hypertension Cases		Controls	
**N or n**	**4,641**		**46,410**		**2,306**		**26,660**		**1,570**		**13,825**		**765**		**5,925**	
	**mean**	**±SD or %**	**mean**	**±SD or %**	**mean**	**±SD or %**	**mean**	**±SD or %**	**mean**	**±SD or %**	**mean**	**±SD or %**	**mean**	**±SD or %**	**mean**	**±SD or %**
**Sociodemographic Characteristics**																
Mean age ± SD, years	55.6	± 8.7	56.6	± 8.5	55.6	± 8.8	56.0	± 8.4	55.1	± 8.6	56.9	± 8.4	58.0	± 8.7	56.6	± 8.7
Age Category																
≤55 years		49.6		45.3		48.8		47.2		53.1		44.8		44.7		38.4
>55 years		50.4		54.7		51.2		52.8		46.9		55.2		55.3		61.6
Region of residence lived longest as an adult																
Northeast		18.9		20.7		20.7		21.7		16.0		18.2		19.3		21.7
Midwest		32.0		29.4		29.4		26.5		34.1		32.7		35.9		35.2
South		29.0		26.3		29.1		26.7		29.5		25.4		27.8		26.4
West		19.8		23.2		20.6		24.6		20.3		23.5		16.6		16.5
Puerto Rico or outside US and Puerto Rico		0.3		0.4		0.3		0.5		0.2		0.2		0.3		0.3
Marital Status																
Married/living as married		78.0		79.1		75.9		77.9		78.0		79.9		84.2		82.9
Single/never married		4.7		3.9		6.5		5.3		3.5		2.2		1.8		1.6
Divorced/separated/widowed		17.3		16.9		17.6		16.8		18.5		17.9		14.0		15.5
Educational attainment																
≤ High school		16.5		12.8		0.0		0.0		0.0		0.0		100.0		100.0
Some college or technical degree		33.8		29.8		0.0		0.0		100.0		100.0		0.0		0.0
≥ College (Bachelor's or greater)		49.7		57.4		100.0		100.0		0.0		0.0		0.0		0.0
Currently employed (yes)		67.4		64.4		70.7		67.4		66.6		62.6		59.1		55.0

© 2023 Gaston SA et al. *JAMA Network Open.*

Annual household income																
Less than $20,000		3.0		2.5		1.3		1.4		4.2		3.5		5.8		5.2
$20,000 to $49,999		21.1		17.9		13.5		11.8		25.6		23.2		35.0		33.3
$50,000 to $99,999		43.5		41.3		41.8		38.5		45.6		45.4		44.2		44.5
$100,000 or more		32.4		38.2		43.4		48.3		24.6		27.9		15.0		16.9
**Health Behaviors**																
Smoking Status																
Current		8.4		6.1		4.9		4.0		11.1		8.2		13.1		19.4
Former		38.9		37.9		36.0		35.5		41.3		40.8		42.5		41.8
Never		52.7		56.1		59.0		60.5		47.5		51.0		44.4		47.8
Alcohol Consumption (past 12 months)																
Current, ≥2 drinks/day		6.4		5.3		7.1		5.5		5.4		5.0		6.4		4.8
Current, <1 to <2 drinks/day		77.8		79.8		80.3		81.9		78.2		78.4		69.4		73.1
Never/Former		15.8		15.0		12.6		12.5		16.4		16.6		24.2		22.1
Log-METs-hours/week ± SD	3.7	± 0.6	3.8	± 0.6	3.7	± 0.6	3.8	± 0.6	3.7	± 0.7	3.8	± 0.6	3.8	± 0.6	3.8	± 0.7
Healthy Eating Index score ± SD	71.4	± 9.7	73.4	± 9.3	73.1	± 9.1	74.6	± 8.8	70.0	± 9.9	72.2	± 9.7	69.1	± 10.4	71.0	± 10.1
Mean sleep score ± SD	1.1	± 1.2	0.9	± 1.2	0.9	± 1.2	0.9	± 1.1	1.1	± 1.3	1.0	± 1.2	1.2	± 1.3	1.2	± 1.2
**Clinical Characteristics**																
BMI category																
Underweight (<18.5 kg/m^2)		0.9		1.5		1.0		1.7		0.8		1.3		0.8		1.3
Recommended weight (18.5-24.9 kg/m^2)		36.2		51.0		41.2		56.3		31.9		45.0		29.9		40.9
Overweight (25.0-29.9 kg/m^2)		35.5		30.8		32.7		28.2		37.6		33.3		39.5		36.8
Obesity (≥30.0 kg/m^2)		27.4		16.7		25.0		13.8		29.7		20.4		29.8		20.9
Postmenopausal (yes)		67.9		71.3		67.9		69.3		66.2		72.8		71.5		77.2
**Racial/ethnic Discrimination**																
Everyday racial/ethnic discrimination																
Yes		4.1		3.6		4.6		4.1		4.1		3.2		2.4		2.1
No		95.9		96.4		95.4		95.9		95.9		96.8		97.6		97.9
Major racial/ethnic discrimination																
Yes		2.7		2.2		3.0		2.4		2.5		2.2		2.4		1.2
No		97.3		97.8		97.0		97.6		97.5		97.8		97.6		98.8

© 2023 Gaston SA et al. *JAMA Network Open.*

Either everyday or major racial/ethnic discrimination								
Yes	6.2	5.2	6.8	5.8	6.1	4.8	4.6	3.2
No	93.8	94.8	93.2	94.2	93.9	95.2	95.4	96.8

Data presented as % or mean ± SD. Percentages may not sum to 100 due to missing values and rounding.

Abbreviations: SD (standard deviation); US (United States); METs (metabolic equivalent of tasks); BMI (body mass index)

Note: Proportion employed is calculated as: number employed/(number employed + unemployed + homemaker + student + retired). Healthy Eating Index scores range from 0-100 with higher scores indicating a healthier diet. Sleep score is a summary score for 6 poor sleep dimensions. Sleep score ranges from 0-6. Participants were assigned a value of one for each if they reported experiencing the following: (1) habitual short [<7-hours] or long [>9-hours] sleep duration (vs. recommended [7-9-hours]); (2) inconsistent weekly sleep patterns, defined as inconsistent wake-up times and bedtimes during the prior six weeks (yes vs. no); (3) sleep debt, defined as ≥2-hour difference between average longest and shortest sleep duration (yes vs. no); (4) frequent napping (≥3 days/week versus <3 days/week); (5) difficulty falling asleep, defined as taking >30 minutes vs. ≤ 30 minutes to fall asleep on average; and (6) difficulty staying asleep, defined as waking up ≥3 times per night ≥3 nights/week vs. <3 times per night <3 nights/week.

Missingness: <0.01% for marital status and BMI category

© 2023 Gaston SA et al. *JAMA Network Open.*

eTable 5. Results of Conditional Logistic Regression: Odds Ratios for Associations Between Reports of Racial and Ethnic Discrimination and Hypertension Incidence, Overall and Stratified by Race and Ethnicity, Sister Study, 2003-2019

Racial/Ethnic Discrimination	n cases/	n controls	n cases/	n controls	OR	95% CI	
**Everyday**	Yes		No				
Overall	500/	4,803	4,679/	46,980	1.14	(1.00,	1.29)
Black/African American	258/	2,502	80/	878	1.23	(0.95,	1.61)
Latina	54/	621	146/	1,372	0.87	(0.63,	1.22)
Non-Hispanic White	188/	1,680	4,453/	44,730	1.17	(1.00,	1.37)
**Major**	Yes		No				
Overall	339/	3,002	4,840/	48,781	**1.29**	**(1.13,**	**1.48)**
Black/African American	183/	1,724	155/	1,656	**1.29**	**(1.03,**	**1.61)**
Latina	29/	278	171/	1,715	1.18	(0.78,	1.79)
Non-Hispanic White	127/	1,000	4,514/	45,410	**1.32**	**(1.09,**	**1.60)**
**Either**	Yes		No				
Overall	626/	4,553	4,553/	46,024	**1.22**	**(1.10,**	**1.36)**
Black/African American	279/	2,676	59/	704	**1.40**	**(1.04,**	**1.88)**
Latina	60/	684	140/	1309	0.87	(0.63,	1.21)
Non-Hispanic White	287/	2,399	4,354/	44,011	**1.26**	**(1.11,**	**1.43)**

Abbreviations: OR (odds ratio); CI (confidence interval)

Models are adjusted for age in 5-year increments, race/ethnicity (in the overall population; Black/African American, Latina, non-Hispanic White), longest lived region of residence (Northeast, Midwest, South, West, Puerto Rico or outside the US and Puerto Rico), educational attainment (≤high school, some college or technical degree, ≥college), and current employment (yes, no). Cases and controls were race/ethnicity- and age-matched.

Bolded values indicate statistical significance at a two-sided p-value of 0.05.

$P_{race/ethnicity*everyday\ racial/ethnic\ discrimination}$=0.23

$p_{race/ethnicity*major\ racial/ethnic\ discrimination}$=0.89

$p_{race/ethnicity*either\ form\ of\ racial/ethnic\ discrimination}$=0.08

© 2023 Gaston SA et al. *JAMA Network Open.*

eTable 6. Results of Conditional Logistic Regression With Additional Adjustment for Potential Mediators: Odds Ratios for Associations Between Reports of Everyday Racial and Ethnic Discrimination and Hypertension Incidence Overall and Within Racial and Ethnic Groups, Stratified by Educational Attainment, Sister Study, 2003-2019

	Everyday racial/ethnic discrimination (no)					Everyday racial/ethnic discrimination (yes)					OR (95% CI); *P* for everyday racial/ethnic discrimination within strata of educational attainment		
	n cases/	n controls	OR	95% CI	*P*	n cases/	n controls	OR	95% CI	*P*			
**Overall**													
≥College	2,283/	26,628	1.00	reference		303/	3,105	**1.19**	**(1.03, 1.38)**	**0.02**	1.04	(0.91, 1.20)	0.55
Some college or technical degree	1,605/	14,136	**1.23**	**(1.14, 1.31)**	**<0.001**	158/	1,380	**1.30**	**(1.07, 1.57)**	**<0.001**	0.93	(0.77, 1.11)	0.40
≤ High school	791/	6,216	**1.33**	**(1.21, 1.46)**	**<0.001**	39/	318	1.29	(0.91, 1.83)	0.15	0.87	(0.62, 1.23)	0.44
**Black/African American**													
≥College	34/	485	1.00	reference		170/	1,621	**1.53**	**(1.04, 2.26)**	**0.03**	**1.53**	**(1.04, 2.26)**	**0.03**
Some college or technical degree	36/	272	**1.79**	**(1.08, 2.96)**	**0.02**	70/	753	1.31	(0.85, 2.03)	0.22	0.73	(0.47, 1.13)	0.16
≤ High school	10/	121	1.02	(0.47, 2.20)	0.96	18/	128	**2.06**	**(1.09, 3.88)**	**0.03**	2.02	(0.87, 4.67)	0.10
**Latina**													
≥College	48/	588	1.00	reference		28/	379	0.94	(0.57, 1.55)	0.81	0.94	(0.57, 1.55)	0.81
Some college or technical degree	64/	488	1.47	(0.97, 2.23)	0.07	23/	178	1.55	(0.90, 2.67)	0.12	1.05	(0.62, 1.77)	0.86
≤ High school	34/	296	1.18	(0.71, 1.96)	0.52	3/	64	0.50	(0.15, 1.72)	0.27	0.43	(0.12, 1.46)	0.17
**Non-Hispanic White**													
≥College	2,201/	27,756	1.00	reference		105/	1,105	1.10	(0.89, 1.35)	0.37	1.10	(0.89, 1.35)	0.37
Some college or technical degree	1,505/	13,376	**1.21**	**(1.13, 1.30)**	**<0.001**	65/	449	**1.54**	**(1.18, 2.01)**	**0.00**	1.27	(0.97, 1.66)	0.08
≤ High school	747/	5,799	**1.35**	**(1.23, 1.48)**	**<0.001**	18/	126	1.38	(0.84, 2.29)	0.20	1.03	(0.62, 1.70)	0.91

Abbreviations: OR (odds ratio); CI (confidence interval); BAA (Black/African American); NHW (non-Hispanic White); RERI (relative excess risk due to interaction)

Models are adjusted for age in 5-year increments, race/ethnicity (in the overall population; Black/African American, Latina, non-Hispanic White), longest lived region of residence (Northeast, Midwest, South, West, Puerto Rico or outside the US and Puerto Rico), annual household income (<$20,000, $20,000-$49,999, $50,000-$99,999, ≥$100,000), smoking status (current, former, never), alcohol consumption (current, ≥2 drinks/day; current, <2 drinks/day; former/never), diet (2015 Healthy Eating Index score), physical activity (metabolic equivalent of tasks hours per week); and poor sleep score (sum of affirmative responses to short or long sleep duration, inconsistent weekly sleep patterns, ≥2 hour difference between shortest and longest sleep duration, napping ≥3 times/week, difficulty falling asleep, difficulty staying asleep). Cases and controls were race/ethnicity- and age-matched.

Note: Bolded values indicate statistical significance at a two-sided p-value=0.05.

Measures of effect modification on the additive scale in the overall population: $RERI_{\text{some college vs. college}}$ = -0.12 (95% CI:-0.39,0.15); $RERI_{\text{high school vs. college}}$ = -0.23 (95% CI:-0.70,0.24)

© 2023 Gaston SA et al. *JAMA Network Open.*

Measures of effect modification on the multiplicative scale in the overall population: ratio of $ORs_{\text{some college vs. college}}$ =0.89 (95% CI:0.72,1.10); ratio of $ORs_{\text{high school vs. college}}$ =0.84 (95% CI:0.58,1.21)

Measures of effect modification on the additive scale among BAA women: $RERI_{\text{some college vs. college}}$ = -1.01 (95% CI:-2.05,0.02); $RERI_{\text{high school vs. college}}$ = 0.51 (95% CI:-0.78,1.79)

Measures of effect modification on the multiplicative scale among BAA women: ratio of $ORs_{\text{some college vs. college}}$ =0.48 (95% CI:0.27,0.85); ratio of $ORs_{\text{high school vs. college}}$ =1.32 (95% CI:0.53,3.31)

Measures of effect modification on the additive scale among Latina women: $RERI_{\text{some college vs. college}}$ = 0.13 (95% CI:-0.78,1.05); $RERI_{\text{high school vs. college}}$ = -0.62 (95% CI:-1.58,0.34)

Measures of effect modification on the multiplicative scale among Latina women: ratio of $ORs_{\text{some college vs. college}}$ =1.12 (95% CI:0.54,2.29); ratio of $ORs_{\text{high school vs. college}}$ =0.45 (95% CI:0.12,1.71)

Measures of effect modification on the additive scale among NHW women: $RERI_{\text{some college vs. college}}$ = 0.23 (95% CI:-0.24,0.70); $RERI_{\text{high school vs. college}}$ = -0.06 (95% CI:-0.79,0.67)

Measures of effect modification on the multiplicative scale among NHW women: ratio of $ORs_{\text{some college vs. college}}$ =1.16 (95% CI:0.82,1.62); ratio of $ORs_{\text{high school vs. college}}$ =0.93 (95% CI:0.54,1.61)

© 2023 Gaston SA et al. *JAMA Network Open.*

eTable 7. Results of Conditional Logistic Regression With Additional Adjustment for Potential Mediators: Odds Ratios for Associations Between Reports of Major Racial and Ethnic Discrimination and Hypertension Incidence Overall and Within Racial and Ethnic Groups, Stratified by Educational Attainment, Sister Study, 2003-2019

	Major racial/ethnic discrimination (no)					Major racial/ethnic discrimination (yes)					OR (95% CI); *P* for major racial/ethnic discrimination within strata of educational attainment		
	n cases/	n controls	OR	95% CI	*P*	n cases/	n controls	OR	95% CI	*P*	OR	95% CI	*P*
**Overall**													
≥College	2,383/	27,752	1.00	reference		203/	1,981	**1.30**	**(1.10, 1.54)**	**<0.001**	1.14	(0.97, 1.33)	0.12
Some college or technical degree	1,662/	14,683	**1.22**	**(1.14, 1.31)**	**<0.001**	101/	833	**1.37**	**(1.10, 1.72)**	**0.01**	0.99	(0.80, 1.23)	0.93
≤ High school	795/	6,346	**1.31**	**(1.20, 1.43)**	**<0.001**	35/	188	**1.99**	**(1.36, 2.89)**	**<0.001**	1.37	(0.94, 1.99)	0.10
**Black/African American**													
≥College	83/	935	1.00	reference		121/	1,171	1.29	(0.95, 1.74)	0.10	1.29	(0.95, 1.74)	0.10
Some college or technical degree	59/	554	1.20	(0.84, 1.73)	0.32	47/	471	1.16	(0.78, 1.71)	0.46	0.96	(0.64, 1.45)	0.86
≤ High school	13/	167	0.83	(0.43, 1.58)	0.57	15/	82	**2.25**	**(1.19, 4.24)**	**0.01**	**2.71**	**(1.20, 6.13)**	**0.02**
**Latina**													
≥College	63/	785	1.00	reference		13/	182	0.95	(0.50, 1.80)	0.88	0.95	(0.50, 1.80)	0.88
Some college or technical degree	73/	604	1.40	(0.97, 2.03)	0.07	14/	62	**2.84**	**(1.44, 5.62)**	**<0.001**	**2.03**	**(1.04, 3.95)**	**0.04**
≤ High school	35/	326	1.15	(0.71, 1.87)	0.56	2/	34	0.55	(0.12, 2.43)	0.43	0.48	(0.11, 2.13)	0.33
**Non-Hispanic White**													
≥College	2,201/	25,555	1.00	reference		105/	1,105	1.25	(0.96, 1.61)	0.09	1.25	(0.96, 1.61)	0.09
Some college or technical degree	1,505/	13,376	**1.22**	**(1.14, 1.31)**	**<0.001**	65/	449	1.36	(0.97, 1.90)	0.07	1.11	(0.79, 1.56)	0.53
≤ High school	747/	5,799	**1.34**	**(1.22, 1.47)**	**<0.001**	18/	126	**2.53**	**(1.50, 4.28)**	**<0.001**	**1.89**	**(1.12, 3.21)**	**0.02**

Abbreviations: OR (odds ratio); CI (confidence interval); BAA (Black/African American); NHW (non-Hispanic White); RERI (relative excess risk due to interaction)

Models are adjusted for age in 5 year increments, race/ethnicity (in the overall population; Black/African American, Latina, non-Hispanic White), longest lived region of residence (Northeast, Midwest, South, West, Puerto Rico or outside the US and Puerto Rico), annual household income (<$20,000, $20,000-$49,999, $50,000-$99,999, ≥$100,000), smoking status (current, former, never), alcohol consumption (current, ≥2 drinks/day; current, <2 drinks/day; former/never), diet (2015 Healthy Eating Index score), physical activity (metabolic equivalent of tasks hours per week); and poor sleep score (sum of affirmative responses to short or long sleep duration, inconsistent weekly sleep patterns, ≥2 hour difference between shortest and longest sleep duration, napping ≥3 times/week, difficulty falling asleep, difficulty staying asleep). Cases and controls were race/ethnicity- and age-matched

Note: Bolded values indicate statistical significance at a two-sided p-value=0.05.

Measures of effect modification on the additive scale in the overall population: $RERI_{\text{some college vs. college}}$ = -0.16 (95% CI:-0.51,0.20); $RERI_{\text{high school vs. college}}$ = 0.37 (95% CI:-0.39,1.13)

Measures of effect modification on the multiplicative scale in the overall population: ratio of $ORs_{\text{some college vs. college}}$ =0.87 (95% CI:0.67,1.13); ratio of $ORs_{\text{high school vs. college}}$ =1.20 (95% CI:0.80,1.80)

Measures of effect modification on the additive scale among BAA women: $RERI_{\text{some college vs. college}}$ = -0.33 (95% CI:-0.96,0.29); $RERI_{\text{high school vs. college}}$ = 1.13 (95% CI:-0.28,2.54)

Measures of effect modification on the multiplicative scale among BAA women: ratio of $ORs_{\text{some college vs. college}}$ =0.75 (95% CI:0.45,1.24); ratio of $ORs_{\text{high school vs. college}}$ =2.10 (95% CI:0.88,5.03)

Measures of effect modification on the additive scale among Latina women: $RERI_{\text{some college vs. college}}$ = 1.49 (95% CI:-0.40,3.37); $RERI_{\text{high school vs. college}}$ = -0.56 (95% CI:-1.71,0.60)

© 2023 Gaston SA et al. *JAMA Network Open.*

Measures of effect modification on the multiplicative scale among Latina women: ratio of ORs $_{\text{some college vs. college}}$ =2.13 (95% CI:0.85,5.35); ratio of ORs $_{\text{high school vs. college}}$ =0.50 (95% CI:0.10,2.58)

Measures of effect modification on the additive scale among NHW women: $RERI_{\text{some college vs. college}}$ = -0.11 (95% CI:-0.66,0.45); $RERI_{\text{high school vs. college}}$ = 0.95 (95% CI:-0.42,2.31)

Measures of effect modification on the multiplicative scale among NHW women: ratio of ORs $_{\text{some college vs. college}}$ =0.89 (95% CI:0.59,1.36); ratio of ORs $_{\text{high school vs. college}}$ =1.52 (95% CI:0.85,2.74)

© 2023 Gaston SA et al. *JAMA Network Open.*

eTable 8. Results of Conditional Logistic Regression With Additional Adjustment for Potential Mediators: Odds Ratios for Associations Between Reports of Either Everyday or Major Racial and Ethnic Discrimination and Hypertension Incidence Overall and Within Racial and Ethnic Groups, Stratified by Educational Attainment, Sister Study, 2003-2019

	Either everyday or major racial/ethnic discrimination (no)					Either everyday or major racial/ethnic discrimination (yes)					OR (95% CI); *P* for major racial/ethnic discrimination within strata of educational attainment	
	n cases/	n controls	OR	95% CI	*P*	n cases/	n controls	OR	95% CI	*P*		
**Overall**												
≥College	2,218/	26,045	1.00	reference		368/	3,688	**1.25**	**(1.09, 1.43)**	**<0.001**	1.10 (0.97, 1.25)	0.14
Some college or technical degree	1,565/	13,850	**1.23**	**(1.15, 1.32)**	**<0.001**	198/	1,666	**1.38**	**(1.16, 1.63)**	**<0.001**	0.99 (0.84, 1.16)	0.86
≤ High school	770/	6,129	**1.32**	**(1.21, 1.45)**	**<0.001**	60/	405	**1.61**	**(1.21, 2.13)**	**<0.001**	1.10 (0.83, 1.47)	0.51
**Black/African American**												
≥College	21/	368	1.00	reference		183/	1,738	**1.95**	**(1.22, 3.12)**	**0.01**	**1.95 (1.22, 3.12)**	**0.01**
Some college or technical degree	30/	222	**2.32**	**(1.29, 4.20)**	**0.01**	76/	76	1.67	(1.00, 2.78)	0.05	0.72 (0.45, 1.14)	0.16
≤ High school	8/	114	1.06	(0.44, 2.56)	0.89	20/	135	**2.75**	**(1.40, 5.38)**	**<0.001**	**2.59 (1.07, 6.26)**	**0.04**
**Latina**												
≥College	47/	560	1.00	reference		29/	407	0.91	(0.55, 1.49)	0.70	0.91 (0.55, 1.49)	0.70
Some college or technical degree	61/	470	1.42	(0.93, 2.17)	0.10	26/	196	1.60	(0.94, 2.72)	0.08	1.12 (0.68, 1.87)	0.65
≤ High school	32/	279	1.18	(0.70, 1.99)	0.53	5/	81	0.61	(0.23, 1.64)	0.33	0.52 (0.19, 1.40)	0.19
**Non-Hispanic White**												
≥College	2,150/	25,117	1.00	reference		156/	1,543	1.17	(0.99, 1.39)	0.07	1.17 (0.99, 1.39)	0.07
Some college or technical degree	1,474/	13,158	**1.21**	**(1.13, 1.31)**	**<0.001**	96/	96	**1.53**	**(1.22, 1.91)**	**<0.001**	**1.26 (1.01, 1.57)**	**0.04**
≤ High school	730/	5,736	**1.34**	**(1.22, 1.47)**	**<0.001**	35/	189	**1.87**	**(1.29, 2.70)**	**<0.001**	1.39 (0.96, 2.02)	0.08

Abbreviations: OR (odds ratio); CI (confidence interval); BAA (Black/African American); NHW (non-Hispanic White); RERI (relative excess risk due to interaction)

Models are adjusted for age in 5 year increments, race/ethnicity (in the overall population; Black/African American, Latina, non-Hispanic White), longest lived region of residence (Northeast, Midwest, South, West, Puerto Rico or outside the US and Puerto Rico), annual household income (<\$20,000, \$20,000-\$49,999, \$50,000-\$99,999, ≥\$100,000), smoking status (current, former, never), alcohol consumption (current, ≥2 drinks/day; current, <2 drinks/day; former/never), diet (2015 Healthy Eating Index score), physical activity (metabolic equivalent of tasks hours per week); and poor sleep score (sum of affirmative responses to short or long sleep duration, inconsistent weekly sleep patterns, ≥2 hour difference between shortest and longest sleep duration, napping ≥3 times/week, difficulty falling asleep, difficulty staying asleep).

Note: Bolded values indicate statistical significance at a two-sided p-value=0.05.

Measures of effect modification on the additive scale in the overall population: $RERI_{\text{some college vs. college}}$ = -0.10 (95% CI:-0.36,0.16); $RERI_{\text{high school vs. college}}$ = 0.03 (95% CI:-0.44,0.50)

Measures of effect modification on the multiplicative scale in the overall population: ratio of $ORs_{\text{some college vs. college}}$ =0.90 (95% CI:0.74,1.09); ratio of $ORs_{\text{high school vs. college}}$ =1.00 (95% CI:0.74,1.36)

Measures of effect modification on the additive scale among BAA women: $RERI_{\text{some college vs. college}}$ = -1.61 (95% CI:-3.16,-0.05); $RERI_{\text{high school vs. college}}$ = 0.74 (95% CI:-0.85,2.33)

Measures of effect modification on the multiplicative scale among BAA women: ratio of $ORs_{\text{some college vs. college}}$ =0.37 (95% CI:0.19,0.71); ratio of $ORs_{\text{high school vs. college}}$ =1.33 (95% CI:0.49,3.60)

© 2023 Gaston SA et al. *JAMA Network Open.*

Measures of effect modification on the additive scale among Latina women: $RERI_{some\ college\ vs.\ college}$ = 0.27 (95% CI:-0.61,1.15); $RERI_{high\ school\ vs.\ college}$ = -0.48 (95% CI:-1.40,0.45)

Measures of effect modification on the multiplicative scale among Latina women: ratio of ORs $_{some\ college\ vs.\ college}$ =1.24 (95% CI:0.62,2.50); ratio of ORs $_{high\ school\ vs.\ college}$ =0.57 (95% CI:0.19,1.73)

Measures of effect modification on the additive scale among NHW women: $RERI_{some\ college\ vs.\ college}$ = 0.14 (95% CI:-0.25,0.53); $RERI_{high\ school\ vs.\ college}$ = 0.35 (95% CI:-0.36,1.07)

Measures of effect modification on the multiplicative scale among NHW women: ratio of ORs $_{some\ college\ vs.\ college}$ =1.07 (95% CI:0.81,1.42); ratio of ORs $_{high\ school\ vs.\ college}$ =1.19 (95% CI:0.79,1.79)

© 2023 Gaston SA et al. *JAMA Network Open.*

eTable 9. Results of Conditional Logistic Regression With Additional Adjustment for Birthplace: Odds Ratios for Associations Between Reports of Everyday, Major, and Either Everyday or Major Racial and Ethnic Discrimination and Hypertension Incidence Among Latina Women, Stratified by Educational Attainment, Sister Study, 2003-2019

	Latina Participants										OR (95% CI); *P* for everyday racial/ethnic discrimination within strata of educational attainment		
**Educational Attainment**	**n cases/**	**n controls**	**OR**	**95% CI**	***P***	**n cases/**	**n controls**	**OR**	**95% CI**	***P***			
	**Everyday racial/ethnic discrimination (no)**					**Everyday racial/ethnic discrimination (yes)**							
≥College	48/	588	1.00	reference		28/	379	0.94	(0.57, 1.55)	0.81	0.94	(0.57, 1.55)	0.81
Some college or technical degree	64/	488	**1.68**	**(1.12, 2.51)**	**0.01**	23/	178	**1.78**	**(1.05, 3.04)**	**0.03**	1.06	(0.63, 1.78)	0.82
≤ High school	34/	296	1.53	(0.95, 2.46)	0.08	3/	64	0.66	(0.20, 2.20)	0.49	0.43	(0.13, 1.46)	0.18
	**Major racial/ethnic discrimination (no)**					**Major racial/ethnic discrimination (yes)**							
≥College	63/	785	1.00	reference		13/	182	0.97	(0.52, 1.83)	0.93	0.97	(0.52, 1.83)	0.93
Some college or technical degree	73/	604	**1.58**	**(1.10, 2.26)**	**0.01**	14/	62	**3.53**	**(1.83, 6.82)**	**<0.001**	**2.23**	**(1.16, 4.30)**	**0.02**
≤ High school	35/	326	1.47	(0.93, 2.30)	0.10	2/	34	0.77	(0.18, 3.32)	0.73	0.53	(0.12, 2.31)	0.40
	**Either everyday or major racial/ethnic discrimination (no)**					**Either everyday or major racial/ethnic discrimination (yes)**							
≥College	47/	560	1.00	reference		29/	407	0.90	(0.55, 1.48)	0.69	0.90	(0.55, 1.48)	0.69
Some college or technical degree	61/	470	**1.62**	**(1.08, 2.44)**	**0.02**	26/	196	**1.82**	**(1.09, 3.06)**	**0.02**	1.12	(0.68, 1.86)	0.65
≤ High school	32/	279	1.52	(0.93, 2.48)	0.10	5/	81	0.81	(0.31, 2.11)	0.66	0.53	(0.20, 1.42)	0.21

Abbreviations: OR (odds ratio); CI (confidence interval); RED (racial/ethnic discrimination)

Models are adjusted for age in 5-year increments, nativity/birthplace (US states, Puerto Rico or outside the US and Puerto Rico), longest lived region of residence (Northeast, Midwest, South, West, Puerto Rico or outside the US and Puerto Rico), and current employment (yes, no). Cases and controls were race/ethnicity- and age-matched.

Note: Bolded values indicate statistical significance at a two-sided p-value=0.05.

Everyday RED - Measures of effect modification on the additive scale: $RERI_{\text{some college vs. college}}$ = 0.16 (95% CI:-0.85,1.17); $RERI_{\text{high school vs. college}}$ = -0.81 (95% CI:-1.97,0.34)

Everyday RED- Measures of effect modification on the multiplicative scale: ratio of $ORs_{\text{some college vs. college}}$ =1.13 (95% CI:0.55,2.30); ratio of $ORs_{\text{high school vs. college}}$ =0.46 (95% CI:0.12,1.70)

Major RED - Measures of effect modification on the additive scale: $RERI_{\text{some college vs. college}}$ = 1.98 (95% CI:-0.28,4.23); $RERI_{\text{high school vs. college}}$ = -0.67 (95% CI:-2.10,0.77)

Major RED - Measures of effect modification on the multiplicative scale: ratio of $ORs_{\text{some college vs. college}}$ =2.30 (95% CI:0.93,5.69); ratio of $ORs_{\text{high school vs. college}}$ =0.54 (95% CI:0.11,2.74)

Everyday or major RED - Measures of effect modification on the additive scale: $RERI_{\text{some college vs. college}}$ = 0.30 (95% CI:-0.67,1.27); $RERI_{\text{high school vs. college}}$ = -0.62 (95% CI:-1.73,0.50)

Everyday or major RED - Measures of effect modification on the multiplicative scale: ratio of $ORs_{\text{some college vs. college}}$ =1.24 (95% CI:0.62,2.50); ratio of $ORs_{\text{high school vs. college}}$ =0.59 (95% CI:0.20,1.77)

© 2023 Gaston SA et al. *JAMA Network Open.*
