# Supplementary material for: Racial and Ethnic Discrimination and Hypertension by Educational Attainment Among a Cohort of US Women
Source: JAMA Netw Open. 2023 Nov 22;6(11):e2344707. doi: 10.1001/jamanetworkopen.2023.44707 (PMC10665977; doi:10.1001/jamanetworkopen.2023.44707)
Supplement: Supplement 2. — Data Sharing Statement [file jamanetwopen-e2344707-s002.pdf]

Data Sharing Statement

Gaston. Racial and Ethnic Discrimination and Hypertension by Educational Attainment Among a Cohort of US Women. *JAMA Netw Open*. Published November 22, 2023. doi:10.1001/jamanetworkopen.2023.44707

Data
Data available: No

Additional Information
Explanation for why data not available: Data, including anonymized data for replication, are available as described on the Sister Study website (https://sisterstudy.niehs.nih.gov/English/coll-data.htm). Data use is restricted to replication, only. New analyses must be approved after a proposal process. Code can be requested from the corresponding author.
